# Direct Colorimetric Temperature Measurement Ahead of Flame Zone with Polydiacetylenes

**DOI:** 10.1021/acsomega.4c11238

**Published:** 2025-03-04

**Authors:** Tanner J. Finney, Abigail W. Wilson, Marisa L. Poveda, Benjamin L. Davis

**Affiliations:** MPA-11: Materials Synthesis and Integrated Devices, Materials Physics and Applications Division, Los Alamos National Laboratory, Los Alamos, New Mexico 87545, United States

## Abstract

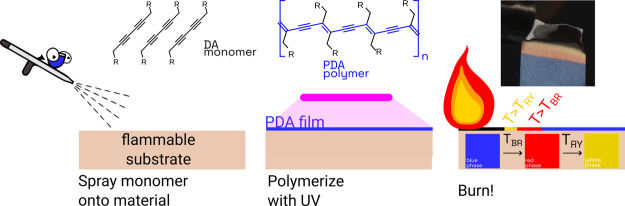

Measurement of temperature and heat transfer ahead of spreading fire is essential to developing our understanding of the mechanisms involved in fire spread and explosions. Because the temperature gradients in spreading fires are often steep and occur on short spatial and time scales, these measurements are notoriously difficult. Current techniques such as thermocouples require painstakingly careful engineering to ensure accurate results. Other approaches such as thermal cameras and Schlieren imaging require expensive, complex optical setups that are expensive, not amenable to widespread deployment, and require specialized expertise to deploy. Polydiacetylenes (PDAs), a class of color-changing polymers, were developed into dynamic temperature sensors, enabling the direct measurement of temperature gradients ahead of a spreading fire. Diacetylene (DA) precursors (polymerized to form PDAs) were synthesized to undergo visible color changes in response to well-defined temperatures ranging from ≈50 to 200 °C. PDAs were coated on flammable substrates, and their responsiveness was demonstrated with the combustion of paper, cardboard, and nitrocellulose. PDAs were found to be able to directly track temperature gradients ahead of the combustion zone with sub-millimeter and microsecond resolution. The developed sensors were demonstrated to have broad applicability due to their ease of deployment, simple readout, and low cost. Dynamic PDA sensors are particularly applicable to situations demanding high spatial and temporal resolutions, such as deflagrations (fast) or backing fires (short-range temperature gradients).

## Introduction

The measurement of heat transfer and temperature gradients ahead of the flame front is key to exploring the mechanisms of fire spread. It is a particular challenge for situations where temperature gradients exist on a very fine spatial or temporal scale and are difficult to isolate from the surrounding environment.^1,2^ Temperature in a spreading fire can be directly measured using thermocouples.^1−6^ For instance, Korobeinichev et al. directly measured the temperature of a burning pine needle with carefully embedded thermocouples.^3,7^ This approach requires precise manufacturing and positioning of the thermocouples to obtain reproducible results. Olson et al. used thermal cameras and infrared measurements to measure fuel heating and flame spread in microgravity.^8^ Optical techniques like interferometry,^9^ Schlieren imaging,^10^ particle imaging velocimetry,^11^ and laser Doppler velocimetry^2^ have also been extensively used to examine heat transfer mechanisms in spreading fires. For instance, Hirano et al. used Schlieren imaging to examine opposed flow combustion and short-range convective heating, and Weber and Mestre used interferometry to measure short-range flame spread rate along fine fuels.^9,10^ Recently Burnford et al. reported the novel use of a thin phosphor coating to directly measure surface temperatures during downward flame spread.^12^ All of these approaches, while yielding a wealth of information about flame spread, require complex instrumentation to achieve reliable results. This work details a complementary technique for simple, direct measurement of surface temperature gradients ahead of a spreading fire with a temperature-sensitive color-changing polymer.

**Figure 1:**
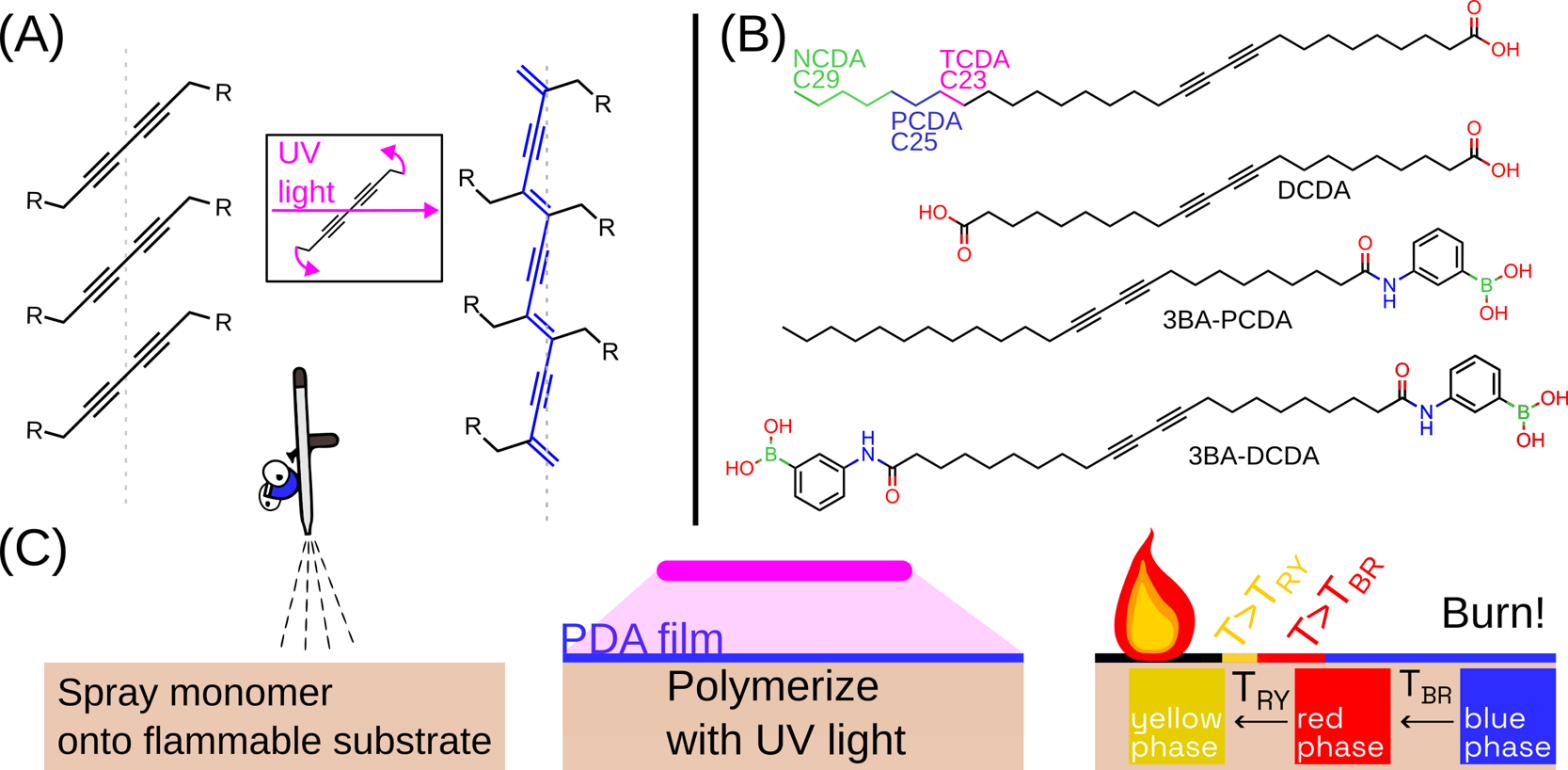
(A) Schematic of topochemical polymerization requirements of diacetylenes into PDA figure is adapted from Wegner^22^ Copyright 2003 John Wiley and Sons, see Enkelmann for more details.^31^ (B) Commercially available DA monomers: PCDA, TCDA, NCDA, and DCDA and their boronic acid functionalized variants synthesized here. (C) Schematic of the operation of the PDA combustion sensor: a flammable material is coated in DA monomers, the monomers are polymerized via UV light and the substrate appears blue, the substrate is ignited, and temperatures are directly read out through the color changes in the polymer film.

Polydiacetylenes (PDAs) are well-known for their vibrant colorimetric transitions.^13,14^ Exposing diacetylene (DA) monomers to UV light induces solid-state (topochemical) polymerization to produce a visibly blue polymer, Figure 1A. This “blue phase” exists as a metastable state. When exposed to sufficient external stimuli, such as heat, the blue phase undergoes a chromatic transition to the red phase, appearing visibly red.^15,16^ Further heating the red phase to higher temperatures induces an additional chromatic transition from red to yellow, creating yellow phase PDA.^17,18^ The mechanism and underlying polymer structure of PDAs remain under active investigation.^19^ However, spectroscopic and structural measurements suggest that the blue phase consists of linear, planar polymers.^19^ Application of external stimuli induces a structural shift, e.g. twisting, in the polymer backbone toward a nonplanar geometry.^20,21^ This shift in the backbone orientation changes the electronic structure, inducing vibrant color changes.^19^

Since their synthesis by Wegner and early demonstrations by Charych et al., PDAs have been investigated for potential sensing applications.^13,14,22−25^ A particular focus has been on lowering the threshold of the color change, driving the sensitivity toward very small stimuli (such as binding to trace metals or biomolecules).^13,26,27^ This work explores an opposing interest in increasing the threshold of color change to dynamically measure high temperatures. While the structure of the different phases is not fully elucidated, it is thought that increasing hydrogen bonding, π-stacking, van der Waals forces, and other intermolecular forces stabilizes the blue phase and increases the stimuli required for the blue-to-red transition.^28,29^ In this work, we designed PDAs with tunable temperature sensitivity ranging from ≈50 to 200 °C and developed them into dynamic temperature sensors with broad applications in combustion and fire spread.

## Materials and Methods

### Diacetylene Monomers

10,12-Tricosadiynoic acid (TCDA), 10,12-pentacosadiynoic acid (PCDA), 10,12-nonacosadiynoic acid, and 10,12-docosadiynedioic acid (DCDA) (Figure 1B) were purchased from TCI America and Fisher Scientific. PCDA and DCDA were functionalized with two different boronic acid head groups (3-aminophenylboronic acid (3BA) and 4-aminophenylboronic acid (4BA)). Boronic acid functionalized DAs^30^ were abbreviated as 4BA-PCDA, 3BA-DCDA, etc. Details of the synthesis and preparation of the monomers are described in the SI, Section S2.

### Calibration and Preparation of PDA Sensors

The temperature response of the PDA was calibrated using a computer-controlled hot plate with a surface-mount thermocouple. A small amount of PDA powder was placed on the hot plate adjacent to the thermocouple and heated in fixed increments. An image was acquired once a steady state temperature was reached. Differential scanning calorimetry (Netzsch Phoenix F204) was used as additional validation of the chromatic transitions. Sensor assembly is straightforward, DA monomers were dissolved and spray coated onto combustible substrates: paper, nitrocellulose, and cardboard. The coated substrates were then exposed to 254 nm UV light until the substrate appeared blue. The substrates were then ignited and temperatures were tracked via a color camera, Figure 1C. Full calibration, sensor preparation, and combustion methods are available in SI Sections S4 and S3.1.

## Results and Discussion

### Calibration of PDA Sensors

The temperature sensitivity of the PDAs was calibrated optically. PDA powders were gradually heated in fixed intervals, and color images were acquired once a steady state temperature was reached. The red, green, and blue channels from each image were monitored as a function of temperature. The blue-to-red transition temperature, *T*_BR_, was identified from a prominent change in the red channel. The red-to-yellow transition temperature, *T*_RY_, was identified by changes in the green and blue channels.

**Figure 2:**
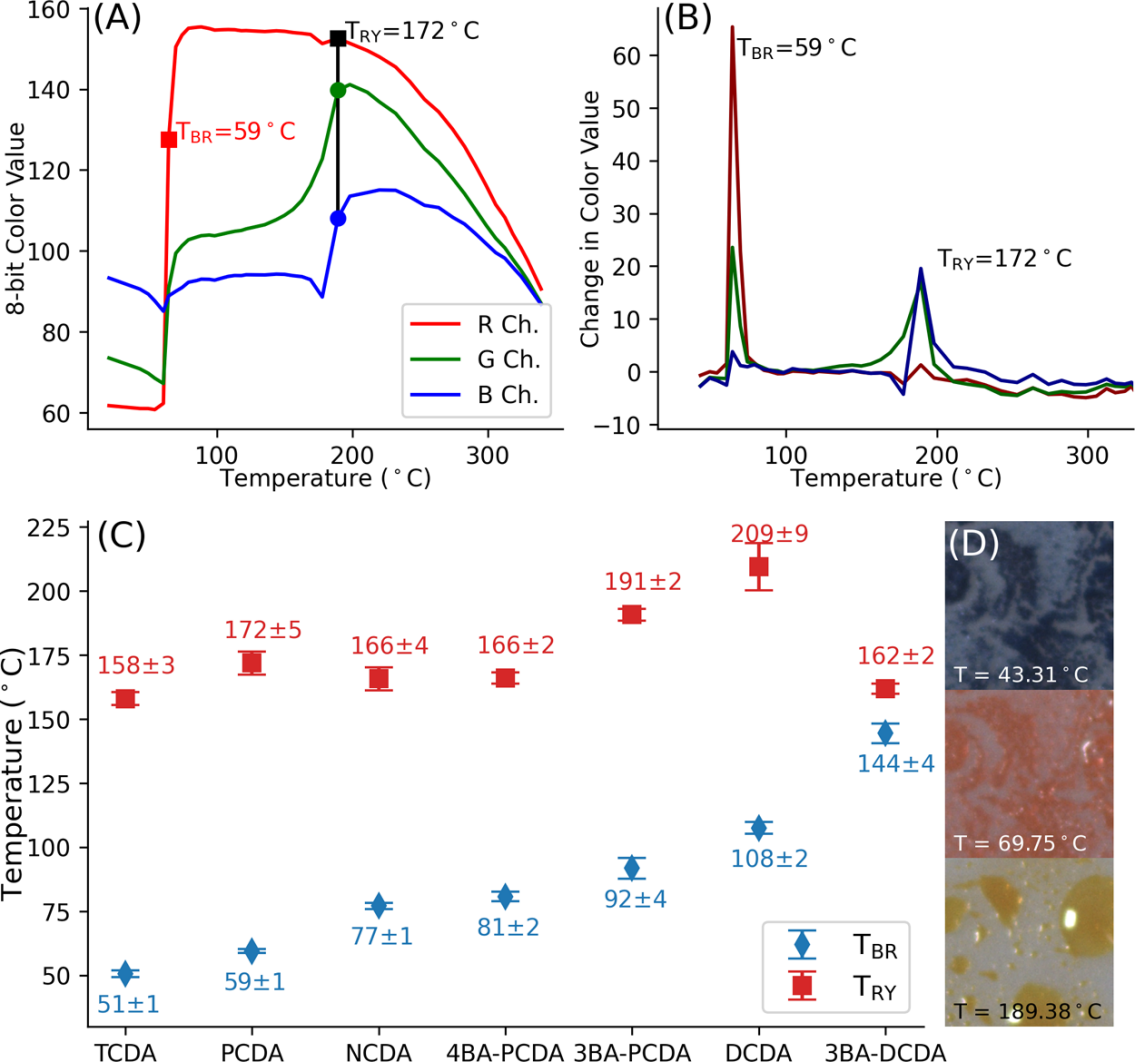
Summary of the calibration method and results for all PDAs are shown in Figure 1. (A) Average RGB values for a uniform area during incremental heating (PCDA used as an example). (B) First derivative of the RGB channels with peaks indicating the blue to red (*T*_BR_), and red to yellow (*T*_RY_) color transitions. (C) *T*_BR_, is tunable from 51 to 145 °C, and *T*_RY_, from 158 to 210 °C. Error bars are one standard deviation from triplicate calibration. (D) Photographs taken with a machine vision camera during calibration of PCDA.

This process was repeated for PDAs made from each DA monomer, as shown in Figure 1B to develop Figure 2. Increasing the chain length of the DA monomer (TCDA → PCDA → NCDA) increases van der Waals attractions between neighboring DA monomers and thus increases *T*_BR_.^28^ Adding a boronic acid headgroup (e.g., PCDA → 4BA-PCDA), which possesses strong hydrogen bonding and π-stacking, was observed to increase *T*_BR_. The highest measured *T*_BR_ (*T* = 144 ± 4 °C) was achieved with a DCDA monomer functionalized with two 3-aminophenylboronic acid head groups (3BA-DCDA). Interestingly there was no increase in *T*_RY_ for 3BA-DCDA. DCDA possessed the highest measured *T*_RY_ (*T* = 209 ± 9 °C). Full calibration methods and data are available in the SI Section S4.

### Temperature Tracking during Fire Spread

**Figure 3:**
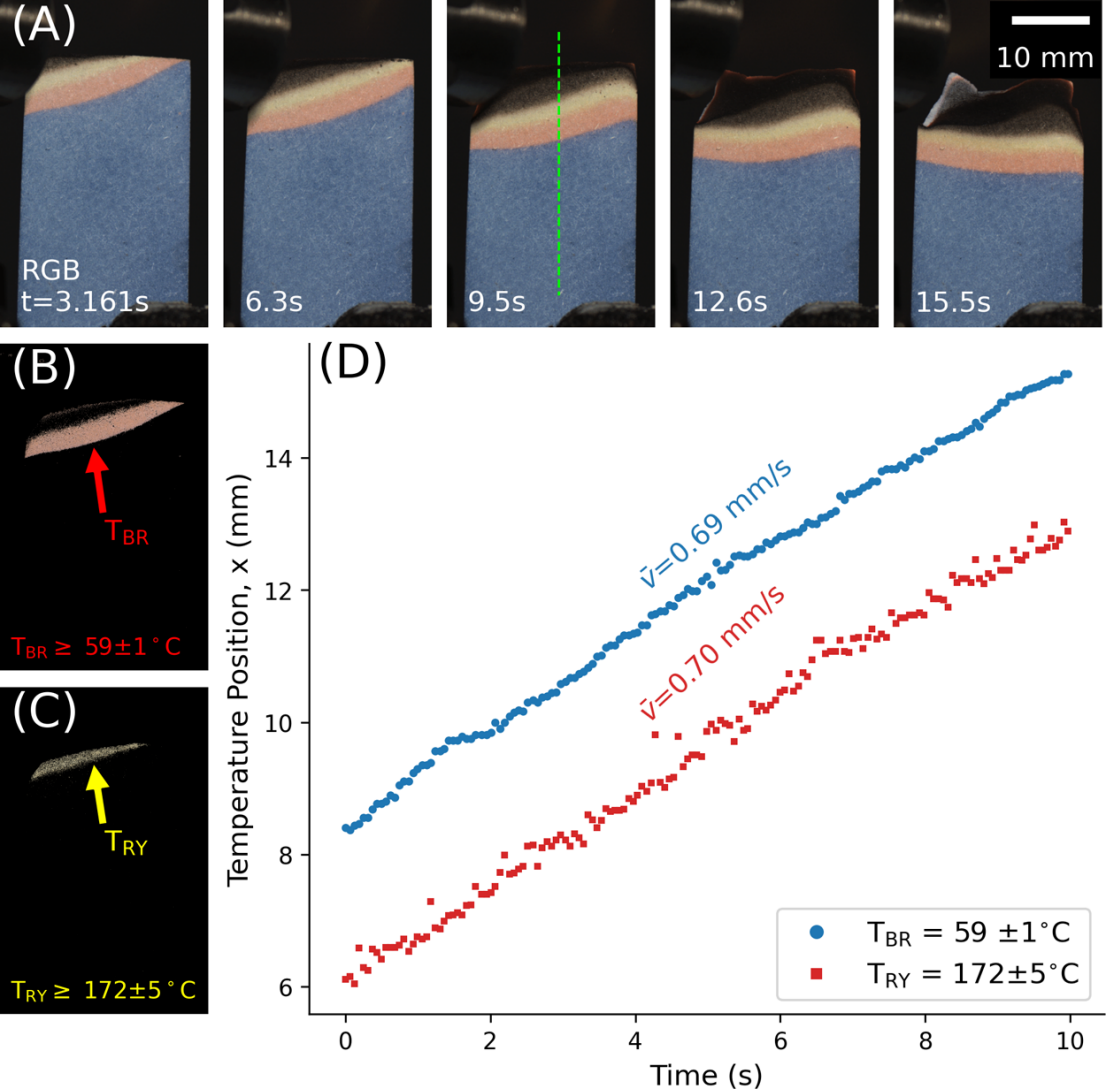
Tracking temperatures via red and yellow phase PDA (PCDA) during combustion using HSV thresholding. (A) Original color images. (B) Thresholded image to track the red phase (*T* ≥ 59 ± 1 °C); the arrow indicates the blue to red transition temperature. (C) Thresholded image to track the yellow phase (*T* ≥ 172 ± 5 °C). The arrow indicates the interface between the yellow and red phase. (D) Direct tracking of the temperature from image thresholding and recording the position of the blue–red and red–yellow interfaces from a vertical image slice (green dashed line in (A)) as a function of time. *v̅* indicates the average velocity () of the two temperatures. This burn is shown in SI Video 1. Full details are shown in SI Section S5.

Figure 3A shows select images during the combustion of PDA-coated paper. A hue saturation value (HSV) threshold was used to extract the red and yellow bands from the RGB image, Figure 3B,C. The interface between the blue and red band corresponded to *T*_BR_ (red arrow in Figure 3B). *T*_RY_ was determined from the interface of the red and yellow bands (yellow arrow in Figure 3C). Once the position of the red and yellow phases is known, the position of *T*_BR_ and *T*_RY_ was directly tracked as the paper burns, Figure 3D. The average separation between *T*_BR_ and *T*_RY_, Δ*x*, was 2.4 ± 0.2 mm. The progression of each band was measured to be linear, in agreement with a flame spread on thermally thin fuels.^1,10,32^

### Combustion of PDA-Coated Cardboard Combs

**Figure 4:**
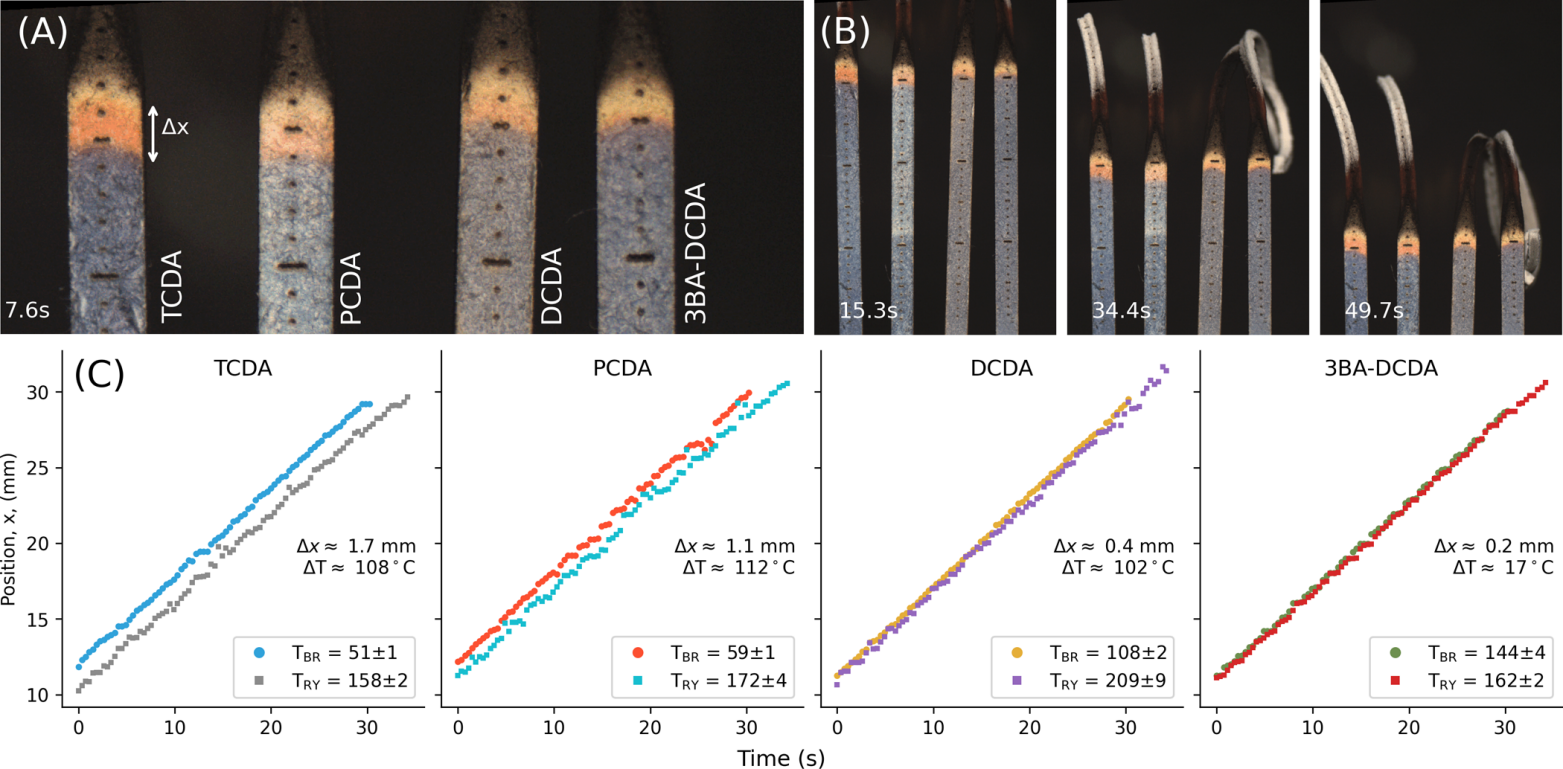
(A) Laser cut cardboard combs with each tine coated in different DAs. Tick marks on the cardboard 1 mm apart. Left to right: TCDA (*T*_BR_ = 51 ± 1 °C and *T*_RY_ = 158 ± 3 °C), PCDA (*T*_BR_ = 59 ± 1 °C and *T*_RY_ = 172 ± 5 °C), DCDA (*T*_BR_ = 108 ± 2 °C and *T*_RY_ = 209 ± 9 °C ), and 3BA-DCDA (*T*_BR_ = 144 ± 4 °C and *T*_RY_ = 162 ± 2 °C). (B) Select frames of PDA combs burning (C) Position over time of the temperature bands during combustion. The calibrated temperature difference, Δ*T*, and separation between the red and yellow bands, Δ*x*, is indicated on each plot. This measurement is shown in SI Video 2.

The cardboard with a white veneer was cut into 3 mm wide combs. They were chosen due to their well-known, uniform, material properties, ease of fabrication and customization, and applicability to wildland fire spread.^33,34^ The tines of laser-cut cardboard combs were coated in DAs with increasing transition temperatures (*T*_BR_ and *T*_RY_) and ignited simultaneously. Figure 4A,B shows select frames from the ignition and combustion of the comb assembly. Each tine of the comb was individually tracked to identify the positions of the blue, red, and yellow phases, Figure 4C. The spread rate of each temperature band was constant between each time, *v̅*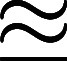 0.58 ± 0.01 mm s^–1^. The average distance between *T*_BR_ and *T*_RY_, Δ*x* was observed to decrease as a function of increasing *T*_BR_ as noted in Figure 4C. Temperatures measured with PDAs were in agreement with previous thermocouple-based measurements of downward flame spread on pine needles which found steep temperature gradients, Δ*T* > 100 °C within millimeters of the flame front.^3^ Likewise, when burning PMMA with phosphor coatings, Burnford et al. found the linear temperature gradient within 2 mm of an opposed flow flame zone to be approximately 124 °C, also demonstrating a steep temperature gradient.^12^ These results are also consistent with Schlieren and interferometric measurements of downward flame spread which demonstrated that fuel heating and convective heat transfer occur on millimeter-length scales.^9,10,32^

### Nitrocellulose Combustion

**Figure 5:**
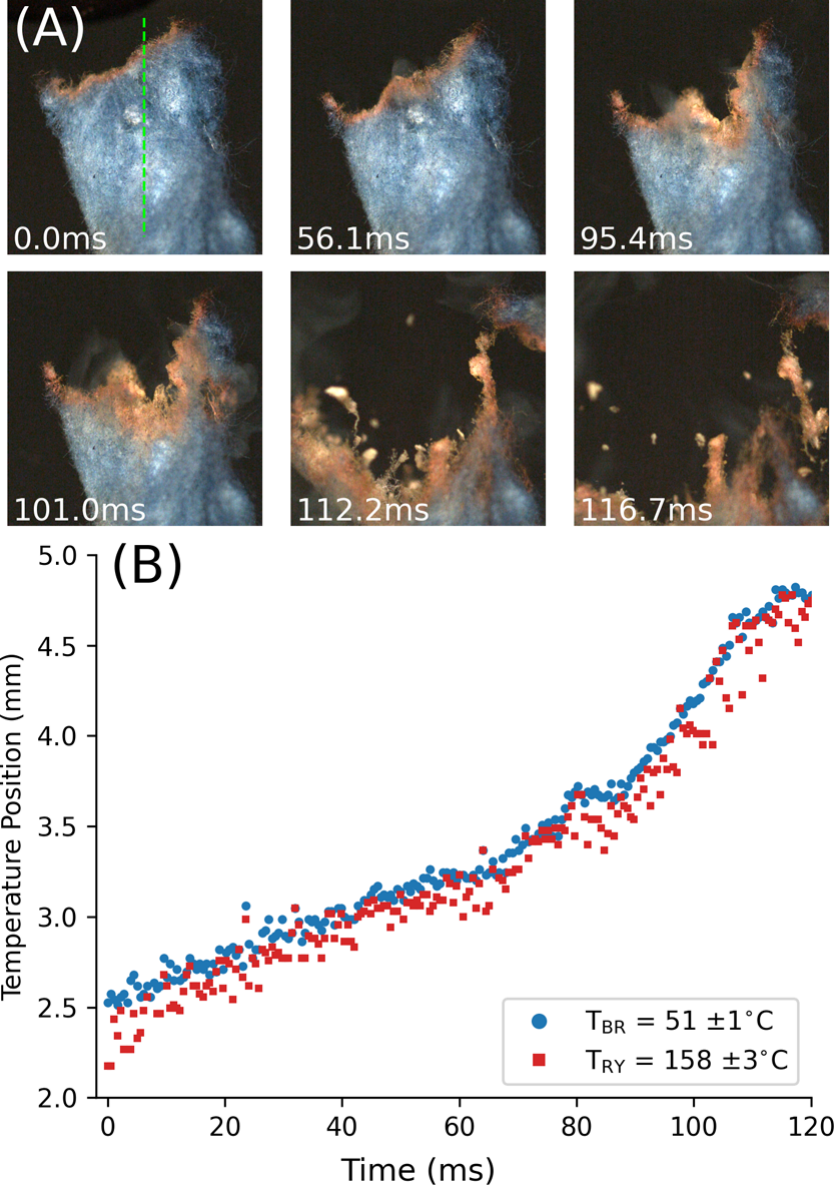
(A) Deflagration of nitrocellulose coated in TCDA, *T*_BR_ = 51 ± 1 °C and *T*_RY_ = 158 ± 3 °C captured at 1700 fps. See SI Video 3. Images are 11 × 11 mm. (B) Tracking the position of *T*_BR_ and *T*_RY_ along the dashed green line. This measurement is shown in SI Video 3.

Nitrated cotton balls were coated in PDAs to investigate the speed of the chromatic transitions. Figure 5A shows still images from the rapid deflagration of nitrocellulose coated in TCDA. Figure 5B shows the positions of *T*_BR_ and *T*_RY_ during combustion along the dashed green line. At approximately 115 ms after ignition, the substrate begins to blow apart. This and further nitrocellulose combustion experiments (shown in SI Section S7) show that PDAs can resolve short-range (submillimeter) temperature gradients in very fast (microsecond) combustion events.

### Applications and Limitations of PDA Sensors

Sensors based on PDAs were found to have a high spatial and temporal resolution. The chromatic transition is thought to be localized and occur at the molecular scale, as shown by previous micro- and nanoscale experiments.^29,35,36^ Unlike other measurement approaches (thermocouples, etc.), the surface temperature of the substrates is directly read out from the color change. While the kinetics of the transition are only modestly investigated, laser spectroscopy experiments by Koshihara et al. measured a phase transition that occurs on the order of 50 ns.^37^ Transition temperatures can be tuned by functionalization of the headgroup, as demonstrated by increasing the blue-to-red transition from 100 to 145 °C with the introduction of boronic acid head groups, significantly higher than commercially available DAs. Combined, this allows for straightforward measurements of temperatures and temperature gradients that occur over short-length scales, like that of buoyant opposed flow combustion and backing fires, or on short time scales such as explosions (potentially both deflagrations and detonations). Additionally, PDA-based sensors may be well suited for investigating heat transfer within smoldering and glowing combustion. A demonstration video of a smoldering PDA-coated incense stick is included in SI Section S9. Once calibrated, PDAs are an inexpensive, easy-to-use addition to the armamentarium of temperature and fire spread measurement techniques. In combination with shadowgraphs, Schlieren imaging, thermal cameras, and thermocouple arrays, PDA sensors could contribute to new insights into a wide range of phenomena within fire science and combustion research.

Strategies to tune the range of PDA temperature sensitivity will be improved by further study of the underlying structures of PDAs in the blue, red, and yellow phases. All PDAs exhibit some degree of chromatic reversibility e.g. red → blue.^26,27,30^ While the DAs developed here are generally irreversible, further investigation into potential reversibility may enrich the temperature resolution of the PDA sensor. Certain PDAs may also exhibit more subtle colors within the blue-to-red and red-to-yellow transitions (purple, orange, etc.). Further precision calibration will enable these to be identified, further enriching the temperature resolution of the current ternary (blue, red, and yellow) approach. Further exploration of the kinetics of the colorimetric transitions is also necessary. While it was demonstrated that PDAs can readily track nitrocellulose deflagrations, further investigation into the color transition rates will enhance the viability of the sensor to quantify heat transfer and temperature spread in explosions and other fast combustion events. A limitation of the currently designed PDA sensors is the maximum temperature sensitivity, currently ≈200 °C, with pyrolysis temperatures often greater than 300 °C. We are currently investigating alternative supramolecular PDA assemblies with higher transition temperatures to record surface temperatures closer to the pyrolysis zone. We hypothesize that temperatures for PDAs are likely sensitive to a maximum temperature of approximately 300 °C and are currently working to report PDA assemblies with these higher transition temperatures in the near future. Follow-up work will also expand the range of flammable substrates tested to include other model materials used in combustion research such as PMMA.

## Conclusions

Polydiacetylenes were developed into dynamic heat transfer sensors with a temperature sensitivity range of ≈50 to 200 °C. PDAs were designed to undergo tunable chromatic transitions at specific, well-defined temperatures. The transition temperatures of PDAs were modified through headgroup functionalization of commercial diacetylene monomers. The sensing properties of the synthesized PDAs were then characterized with several model experiments, including opposed flow combustion of paper and cardboard and rapid combustion of nitrocellulose. These measurements demonstrate that a PDA-based sensor can directly measure the temperature ahead of the combustion zone, revealing steep short-range temperature gradients in opposed flow combustion, consistent with previous thermocouple, Schlierien, and interferometric measurements. Dynamic PDA temperature sensors show potential broad applicability in all aspects of fire spread and combustion research due to their ease of calibration, deployment, and readout.

## Data Availability

All experimental data is available in the Supporting Information. Analysis code and examples are available at 10.5281/zenodo.13913280.

## Supporting Information Available

Video of measurement shown in Figure 3 (MP4)
Video of measurement shown in Figure 4 (MP4)
Video of measurement shown in Figure 5 (MP4)
Complete experimental details, in depth descriptions of synthesis, calibration, experimental procedures, and data analysis (PDF)
