# Supplementary material for: Direct Colorimetric Temperature Measurement Ahead of Flame Zone with Polydiacetylenes
Source: ACS Omega. 2025 Mar 4;10(10):10594–600. doi: 10.1021/acsomega.4c11238 (PMC11923839; doi:10.1021/acsomega.4c11238)
Supplement: Supplementary file 4 — ao4c11238_si_004.pdf [file ao4c11238_si_004.pdf]

Supporting Information for:

Direct Colorimetric Temperature Measurement Ahead of Flame Zone with Polydiacetylenes

Tanner J. Finney*, Abigail W. Wilson, Marisa L. Poveda, Benjamin L. Davis

MPA-11: Materials Synthesis and Integrated Devices, Materials Physics and Applications Division, Los Alamos National Laboratory, New Mexico 87545, United States

*Corresponding Author: Tanner J. Finney

Email: tjfinney@lanl.gov

Contents

S1 Nitrocellulose Synthesis **S4**

S2 Diacetylene Synthesis **S4**

S2.1 Synthesis Overview S4

S2.2 Synthesis of 4-(10,12-pentacosadiynamido) phenylboronic acid (4BA-PCDA) S5

S2.3 Synthesis of 3-(10,12-pentacosadiynamido) phenylboronic acid (3BA-PCDA) S6

S2.4 Synthesis of (((10,12-docosadiynedioyl)bis(azanediyl))bis(3-phenylboronic acid)) (3BA-DCDA) S7

S2.5 Synthesis of (((10,12-docosadiynedioyl)bis(azanediyl))bis(4-phenylboronic acid)) (4BA-DCDA) S8

S2.6 Unsuccessful Diacetylenes S9

S2.6.1 5,7-Dodecadiynedioic acid (DDDA) S10

S2.6.2 3-BA functionalized DDDA S10

S2.7 Comparison of 3BA- and 4BA- functionalization S10

S3 Schematic of Dynamic PDA Temperature Sensors **S11**

S3.1 Preparation of PDA sensors S11

S4 Calibration of PDA Sensors: Optical & DSC methods **S12**

S4.1 Calibration Overview S12

S4.2 Differential Scanning Calorimetry S13

S4.3 Individual DA calibration results S14

S5 Temperature Tracking with PDAs **S16**

S5.1 Combustion Measurements S16

S5.2 Tracking a very fine temperature gradient, 3BA-DCDA S18

S6 Demonstration: Paper Combustion **S19**

S6.1 Paper coated in DCDA S19
S6.2 Opposed Flow Horizontal Combustion: PCDA S20
S6.3 Opposed Flow Horizontal Combustion: DCDA S21
S6.4 Opposed Flow Horizontal Combustion: 3BA-DCDA S22

S7 Demonstration: Nitrocellulose **S22**

S7.1 Guncotton coated in PCDA S23
S7.2 Nitrated Paper coated in PCDA S24
S7.3 Nitrated Paper coated in DCDA S25
S7.4 Nitrated Paper coated in 3BA-DCDA S26

S8 Demonstration: Comb Combustion **S26**

S8.1 Temperature as a Function of Approximate Location S27
S8.2 Horizontal Combustion S27

S9 Demonstration: Smoldering **S28**

S9.1 Incense Stick S28

S10 ^{1}H NMR of each synthesized diacetylene **S28**

S1 Nitrocellulose Synthesis

70% Nitric Acid (Trace metal grade, Fisher Scientific) was slowly added to chilled sulfuric acid (1:2 volume ratio) (99% Sigma Aldrich), and cooled to 0 °C. Once cooled, cotton balls, 100% cotton paper and other cellulosic materials were added to the acid mixture, weighed down and allowed to react for 5 hours. The samples were then removed, neutralized with saturated potassium carbonate (K_2CO_3), washed thoroughly with water and then dried overnight. K_2CO_3 was chosen over the more frequently used sodium bicarbonate to mitigate the intensity of the flame during measurements. Synthesis was verified by auditory testing: the satisfying “fwomp” sound was heard during ignition of the nitrocellulose.

S2 Diacetylene Synthesis

S2.1 Synthesis Overview

All syntheses were conducted with dry glassware under argon atmosphere. Purified diacetylene was dissolved in dry THF, cooled to 0°C, and the appropriate equivalents of oxalyl chloride (TCI America) were added dropwise. The reaction mixture was allowed equilibrate before a drop of dry N,N dimethylformamide (DMF) was introduced, followed by removal of the ice bath, and continuous stirring for 1.5 hours. The reaction was then concentrated to a yellow solid under vacuum to remove residual oxalyl chloride and then redissolved in dry THF.

In a separate flask, appropriate equivalents of an m-aminophenylboronic acid (Boron Molecular) and triethylamine were dissolved in dry THF and cooled to 0°C. The chlorinated diacetylene solution was added dropwise to this mixture. The reaction was allowed to stir overnight under inert atmosphere, then transferred to a large beaker. Deionized (DI) water was added slowly to induce precipitation, and the mixture was cooled to 0 °C for 3 hours. White solids were isolated by filtration, washed with DI water and ethyl acetate, and dried under vacuum to yield the desired boronic acid functionalized diacetylene monomer. Products

were characterized with proton NMR and Mass Spectrometry. NMR spectra were acquired on a Bruker AV500 MHz NMR in DMSO-d6. Electrospray ionization (ESI) mass spectra were measured by a Waters Acquity H-Class and a single quadrupole Waters SQ Detector 2 mass spectrometer. Purity and identity were assessed using a Waters Acquity BEH C-18 analytical column (1.7 µm 50 mm x 2.1 mm) at a flow rate of 0.500 $mL\,min^{-1}$ with 0.01% formic acid in water and 0.01% formic acid in methanol as the mobile phases.

S2.2 Synthesis of 4-(10,12-pentacosadiynamido) phenylboronic acid (4BA-PCDA)

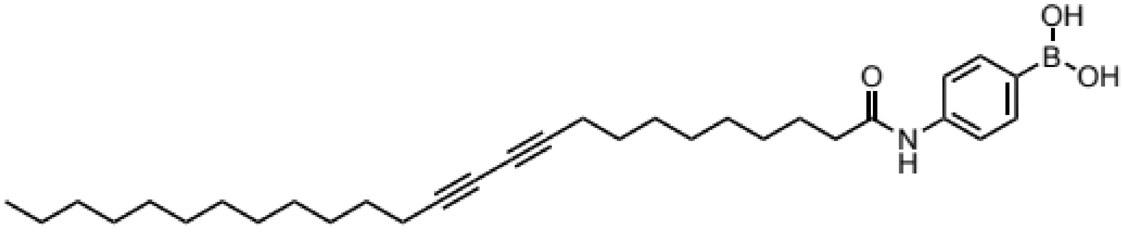

Figure S1: 4-(10,12-pentacosadiynamido) phenylboronic acid (4BA-PCDA)

To a 50 mL flask, in the dark, under argon was added purified 10,12-pentacosadiynoic acid (PCDA) (0.2007g, 0.5358 mmol), dissolved in dry THF (7mL), and cooled to 0 °C. Oxalyl chloride (0.220 mL, 2.565 mmol, 5 eq.) was added, dropwise. The reaction mixture was allowed to stir for 5 minutes before a drop of DMF was introduced, followed by removal of the ice bath, and continuous stirring for 1.5 hours. The reaction was then concentrated under reduced pressure to remove solvent and residual oxalyl chloride, producing a light orange solid. The reactivity of 10,12-pentacosadiynoyl chloride inhibited characterization of the products of this step. The products were redissolved in dry THF (7mL).

In a separate flask under argon, 4-aminophenylboronic acid (0.085 g, 0.4912 mmol, 1.1 eq.) was dissolved in dry THF (8mL). Triethylamine (0.298mL, 2.138 mmol, 4 eq.) was added to the flask and the mixture was cooled to 0 °C. The 10,12-pentacosadiynoyl chloride solution

was then added, dropwise, and allowed to stir overnight, in the dark, under argon, at room temperature. The following day, DI water (85mL) was added to the reaction flask, causing rapid precipitation of white solids. Solids were isolated by filtration, washed with DI water and ethyl acetate, and dried under vacuum to yield 4-(10,12-pentacosadiynamido) phenylboronic acid (0.1717 g, 0.3479 mmol, 65% yield) as a white solid.

^{1}H NMR (500 MHz, DMSO) δ 9.85 (s, 1H), 7.86 (s, 2H), 7.69 (d, 2H), 7.53 (d, J = 8.3 Hz, 2H), 2.28 (dt, J = 11.8, 7.2 Hz, 6H), 1.58 (t, J = 7.2 Hz, 2H), 1.49 - 1.38 (m, 4H), 1.36 - 1.18 (m, 26H), 0.85 (t, J = 6.9 Hz, 3H).

S2.3 Synthesis of 3-(10,12-pentacosadiynamido) phenylboronic acid (3BA-PCDA)

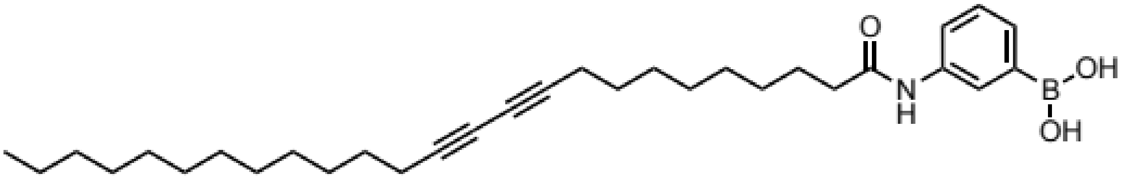

Figure S2: 3-(10,12-pentacosadiynamido) phenylboronic acid (3BA-PCDA)

To a 50 mL flask, in the dark, under argon was added purified 10,12-pentacosadiynoic acid (PCDA) (0.403g, 1.076 mmol), dissolved in dry DCM (7mL), and cooled to 0°C. Oxalyl chloride (0.230 mL, 2.682 mmol, 2.5 eq.) was added, dropwise. The reaction mixture was allowed to stir for 5 minutes before a drop of DMF was introduced, followed by removal of the ice bath, and continuous stirring for 2 hours. The reaction was then concentrated under reduced pressure to remove solvent and residual oxalyl chloride, producing a light orange solid. The reactivity of 10,12-pentacosadiynoyl chloride inhibited characterization of the products of this step. The products were redissolved in dry THF (9mL).

In a separate flask under argon, 3-aminophenylboronic acid (0.3105 g, 1.7906 mmol, 2.1 eq.) was dissolved in dry THF (10mL). Triethylamine (0.600mL, 4.305 mmol, 4 eq.) was added to

the flask and the mixture was cooled to 0°C. The 10,12-pentacosadiynoyl chloride solution was then added, dropwise, and allowed to stir overnight, in the dark, under argon, at room temperature.

The following day, DI water was added to the reaction flask, causing rapid precipitation of white solids. Solids were isolated by filtration, washed with DI water and ethyl acetate, and dried under vacuum to yield 3-(10,12-pentacosadiynamido) phenylboronic acid (0.0974 g, 0.1973 mmol, 18% yield) as a white solid.

^{1}H NMR (500 MHz, DMSO) δ 9.76 (s, 1H), 7.97 (s, 2H), 7.81 (s, 1H), 7.70 (d, J = 2.1 Hz, 1H), 7.45 (d, J = 7.3 Hz, 1H), 7.23 (t, J = 7.7 Hz, 1H), 2.27 (td, J = 7.1, 4.4 Hz, 6H), 1.58 (t, J = 7.2 Hz, 2H), 1.49 - 1.39 (m, 4H), 1.34 - 1.20 (m, 27H), 0.85 (t, J = 6.8 Hz, 3H).

Poor yield for this reaction can be attributed to the significant impurities in this batch of 3-aminophenylboronic acid starting material, and use of triethylamine which caused poor yield for the meta position boronic acid.

S2.4 Synthesis of (((10,12-docosadiynedioyl)bis(azanediyl))bis(3-phenylboronic acid)) (3BA-DCDA)

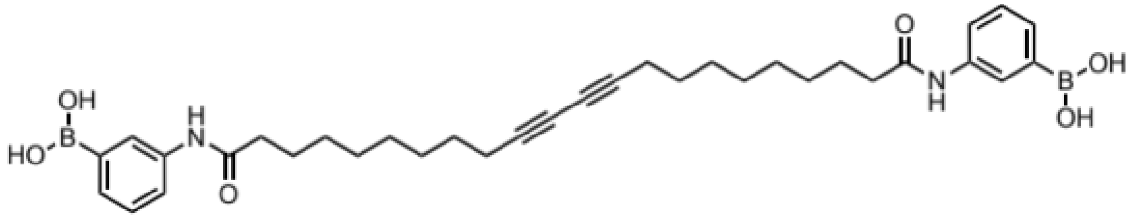

Figure S3: (((10,12-docosadiynedioyl)bis(azanediyl))bis(3-phenylboronic acid)

To a 50 mL flask, in the dark, under argon was added 10,12-docosadiynedioic acid (DCDA) (0.203g, 0.5600 mmol), dissolved in dry THF (7mL), and cooled to 0°C. Oxalyl chloride (0.24 mL, 2.7984 mmol, 5 eq.) was added, dropwise. The reaction mixture was allowed to stir for 5 minutes before a drop of DMF was introduced, followed by removal of the ice bath, and

continuous stirring for 1.5 hours. The reaction was then concentrated under reduced pressure to remove solvent and residual oxalyl chloride, producing a light yellow solid. The reactivity of 10,12-docosadiynedioyl dichloride inhibited characterization of the products in this step. The products were redissolved in dry THF (8mL).

In a separate flask under argon, 3-aminophenylboronic acid (0.318 g, 1.834 mmol, 4.1 eq.) was dissolved in dry THF (10mL) and the mixture was cooled to 0 °C. The 10,12-docosadiynedioyl dichloride solution was then added, dropwise, and allowed to stir overnight, in the dark, under argon, at room temperature.

The reaction contents were transferred to a large beaker and DI water (250mL) was added to the mixture, causing precipitation of white solids. The mixture was allowed to cool for 3 hours at 0°C. Solids were isolated by filtration, washed with DI water and ethyl acetate, and dried under vacuum to yield (((10,12-docosadiynedioyl)bis(azanediyl))bis(3-phenylboronic acid)) (0.242 g, 0.4031 mmol, 72% yield) as a white solid.

^{1}H NMR (500 MHz, DMSO) δ 9.76 (s, 2H), 7.97 (s, 4H), 7.81 (s, 2H), 7.70 (d, 2H), 7.45 (d, J = 1.2 Hz, 2H), 7.23 (t, J = 7.7 Hz, 2H), 2.27 (td, J = 7.1, 4.4 Hz, 8H), 1.57 (t, J = 6.9 Hz, 4H), 1.44 (p, J = 6.9 Hz, 4H), 1.36 - 1.22 (m, 17H).

S2.5 Synthesis of (((10,12-docosadiynedioyl)bis(azanediyl))bis(4-phenylboronic acid)) (4BA-DCDA)

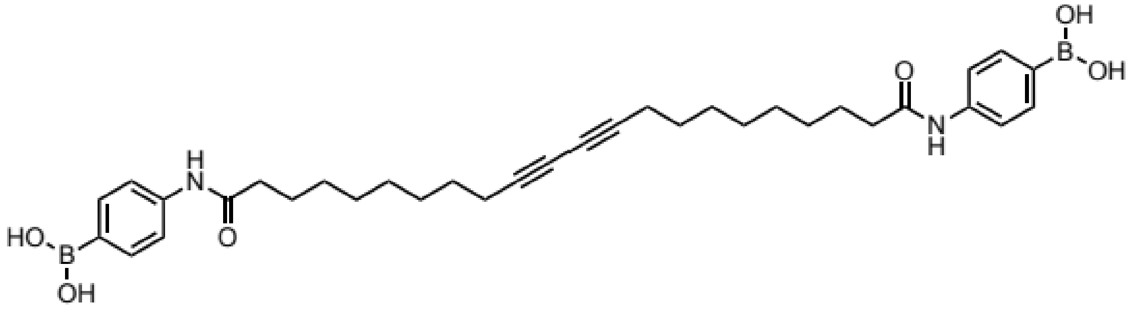

Figure S4: (((10,12-docosadiynedioyl)bis(azanediyl))bis(4-phenylboronic acid)

To a 50 mL flask, in the dark, under argon was added purified 10,12-docosadiynedioic acid (DCDA) (0.1795 g, 0.4952 mmol), dissolved in dry THF (7mL), and cooled to 0°C. Oxalyl chloride (0.210 mL, 2.449 mmol, 5 eq.) was added, dropwise. The reaction mixture was allowed to stir for 5 minutes before a drop of DMF was introduced, followed by removal of the ice bath, and continuous stirring for 1.5 hours. The reaction was then concentrated under reduced pressure to remove solvent and residual oxalyl chloride, producing a light yellow solid. The reactivity of 10,12-docosadiynedioyl dichloride inhibited characterization of the products of this step. The products were redissolved in dry THF (7mL).

In a separate flask under argon, 4-aminophenylboronic acid (0.150 g, 0.8650 mmol, 2.1 eq.) was dissolved in dry THF (8mL). Triethylamine (0.550mL, 3.9460 mmol, 8 eq.) was added to the flask and the mixture was cooled to 0°C. The 10,12-docosadiynedioyl dichloride solution was then added, dropwise, and allowed to stir overnight, in the dark, under argon, at room temperature.

The reaction contents were transferred to a large beaker and DI water (200mL) and ethyl acetate (100 mL) was added to the mixture, causing slight precipitation of white solids. The mixture was allowed to cool for 3 hours at 0°C. Solids were isolated by filtration, washed with DI water and ethyl acetate, and dried under vacuum to yield (((10,12-docosadiynedioyl)bis(azanediyl))bis(4-phenylboronic acid)) (0.2208 g, 0.3678 mmol, 74% yield) as a white solid.

^{1}H NMR (500 MHz, DMSO) δ 9.85 (s, 2H), 7.86 (s, 4H), 7.69 (d, 4H), 7.53 (d, 4H), 2.28 (dt, J = 11.9, 7.1 Hz, 8H), 1.58 (t, J = 6.9 Hz, 4H), 1.44 (p, J = 6.9 Hz, 4H), 1.36 - 1.21 (m, 16H).

S2.6 Unsuccessful Diacetylenes

4-BA DCDA was succesfully synthesized, however polymerization drives the monomer directly into the red phase. While heating/combustion did induce a red to yellow transition, T_{RY} was

approximately 150 °C, which was not remarkable relative to other DAs synthesized here.

S2.6.1 5,7-Dodecadiynedioic acid (DDDA)

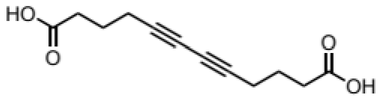

Figure S5: 5,7-Dodecadiynedioic acid (DDDA)

DDDA was obtained from BLDPharm as a brown solid. Upon exposure to UV light, the powder appeared moderately purple-red. Heating induced a weak red to yellow transition at approximately 150 °C. DDDA was not investigated further due to a lack of vibrant color changes.

S2.6.2 3-BA functionalized DDDA

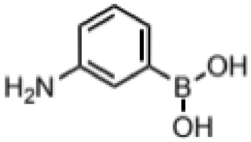

Figure S6: 3-aminophenylboronic acid

Synthesis was largely unsuccessful. DDDA was completely insoluble in dry THF, DCM, acetonitrile, and ethyl acetate. Notably, DDDA dissolved in "wet" THF, but this was not suitable for air and water sensitive synthesis. No product was recovered.

S2.7 Comparison of 3BA- and 4BA- functionalization

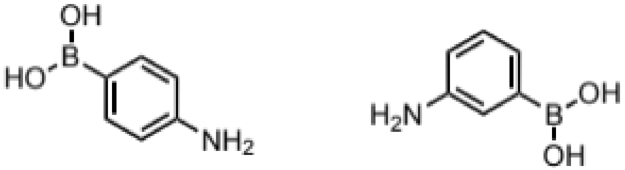

Figure S7: Comparison of 3BA- and 4BA- diacetylenes

3-aminophenylboronic acid functionalized DCDA (3BA-DCDA) clearly displayed the highest temperature threshold for the resulting blue-to-red color change. However, the same DCDA molecule with 4BA functionalization, skipped the blue phase entirely, suggesting the threshold was much lower, or nonexistent.

PCDA functionalized with both meta and para isomers also displayed differences in the color-change threshold. 3BA functionalized PCDA displayed the blue-to-red transition at 10 °Chigher, and 25 °C higher for red to yellow. While this difference is less extreme when compared to DCDA, it does suggest that the meta position increases the threshold of the color change. This is most likely a result of the interactions between the head groups on these molecules. Further investigation is necessary to deduce a mechanism.

S3 Schematic of Dynamic PDA Temperature Sensors

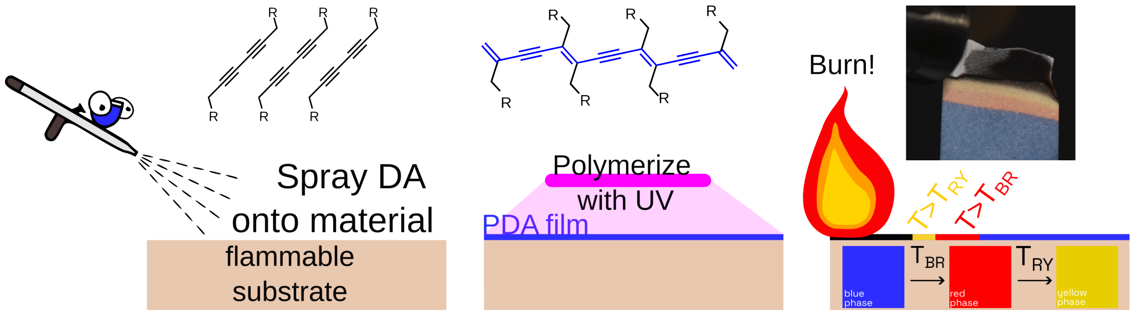

Figure S8: Graphic Abstract/Schematic of PDA sensors for combustion & fire.

S3.1 Preparation of PDA sensors

Three combustible substrates were investigated, 100% cotton paper (Southworth 23 lb), nitrocellulose (synthesis details: section S1) (both cotton balls and cotton paper), and white cardboard (LETTRA 220 lb). White cardboard was laser cut (Universal CO_2 laser cutter) into 3mm wide tines with 1mm markings etched into the cardboard. DA monomers were dissolved in a suitable solvent prior to deposition on a substrate. Chloroform (ethanol stabilized, Fisher

Scientific) or Diethyl ether (HPLC grade, Fisher Scientific) was used for TCDA, PCDA and NCDA. A 30:70% ratio (by volume) of methanol (HPLC grade, Fisher Scientific) to chloroform was used for DCDA, and boronic acid functionalized DAs. A concentration of 30-60 $mg\,mL^{-1}$ of DA in solvent was targeted, though precise concentrations were unnecessary, as long as sufficient monomer was present to ensure polymerization. The DA solution was filtered and then loaded into a battery powered airbrush and sprayed over the desired substrate. Once sprayed, the DA-coated substrates then dried and exposed to 254 nm UV light to induce polymerization. TCDA, PCDA and NCDA took between 30 seconds to 1 minute of low intensity light (UVP-UVGL-58) to become sufficiently polymerized. DCDA and boronic acid functionalized DAs, e.g. 3BA-DCDA, coatings took approximately 3 minutes of high intensity light (UVP Pen-Ray 11SC-1) to become deep blue.

S4 Calibration of PDA Sensors: Optical & DSC methods

The temperature response of PDA powders was measured using an externally controlled hot plate (IKA CMAG-7) with surface mount thermocouple (Omega 88107E). A small amount of PDA powder was placed on the hot plate adjacent to the thermocouple. The PDA powders were heated in fixed increments and once a steady state temperature was reached, an image was acquired using a FLIR BFS-U3-63S4C-C camera (60 fps, 6.3 MP) with 75 mm DG lens and the process was repeated for temperatures between 40°C and 350 °C. A Netzsch Phoenix F204 Differential Scanning Calorimeter (DSC) was used as additional validation of the blue to red phase transition.

S4.1 Calibration Overview

Fig. S9 shows the optical temperature calibration of PDA powders. PDA powders were gradually heated in fixed intervals and images were acquired at each temperature once steady

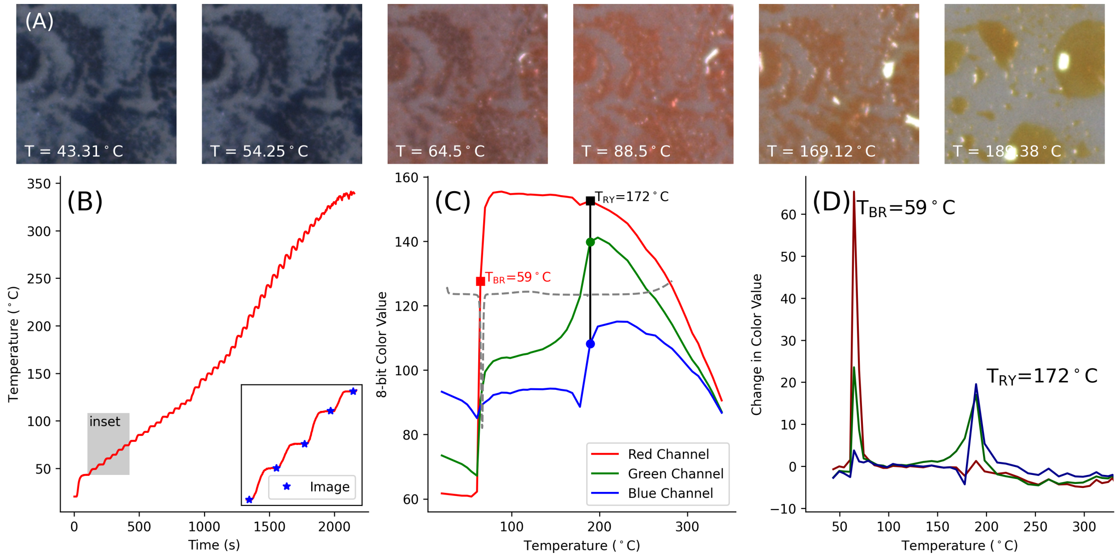

Figure S9: Calibration method for PDA temperature sensors (PCDA as an example) (A) Pictures taken with machine vision camera (B) Temperature is increased in 5 °C increments and the steady state temperature is measured using a PID controlled hot plate, inset: zoomed view of temperature curve showing where images were taken (blue stars). (C) Average RGB values for a uniformly coated area are chosen, the dashed grey indicates a DSC curve of the PCDA monomer. (D) First derivative of the RGB channels with peaks indicating the blue to red (T_{BR}), and red to yellow (T_{RY}) color transitions.

state was reached, Fig. S9B. The red, green and blue channels from each image were then monitored as a function of temperature, Fig. S9C. A sharp change the red channel was observed for the blue to red transition, T_{BR}, and in the green & blue channels for T_{RY}, Fig. S9D. These peaks indicate the temperature at which the material undergoes a chromatic transition.

S4.2 Differential Scanning Calorimetry

4-5 mg of diacetylene powder was the added to a Netzsch low pressure crucible, exposed to UV light and crimped. The crucible was then loaded into a Netzsch 204 F1 Phoenix DSC. The black curves in the following figures are the DSC curves, which were used to examine the blue to red transition, as it is close related to the melting point of the DA monomers. Insufficient polymer content was present to get reliable calorimetry from the red to yellow transition.

Optical calibration was carried out through the same approach as described in the main text, with calibration repreated in triplicate (one example for each material is shown below).

S4.3 Individual DA calibration results

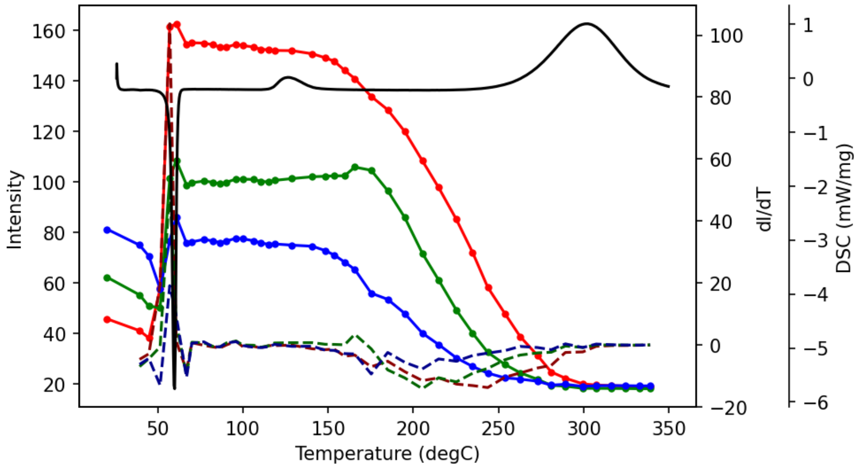

Figure S10: Calibration of 10,12 tricosadiynoic acid (TCDA). The solid lines with 'o' markers are the RGB channels, dashed lines indicate derivatives of the RGB channels. The solid black line is the DSC curve.

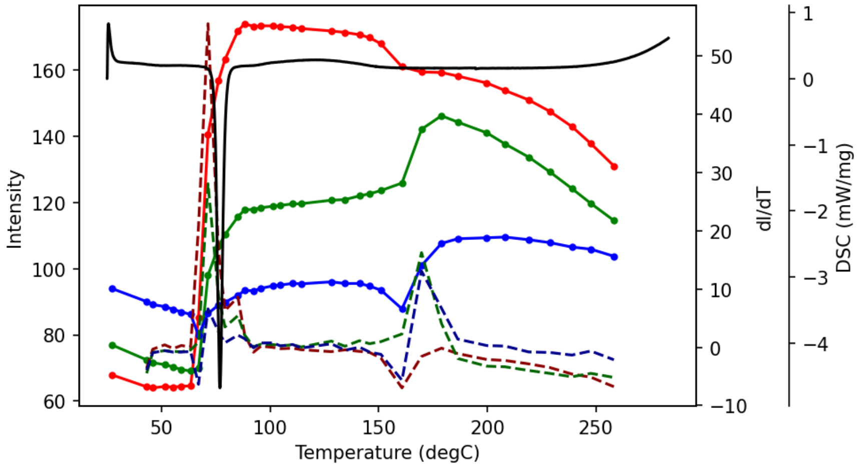

Figure S11: Calibration of 10,12 noncosadiynoic acid (NCDA). The solid lines with 'o' markers are the RGB channels, dashed lines indicate derivatives of the RGB channels. The solid black line is the DSC curve.

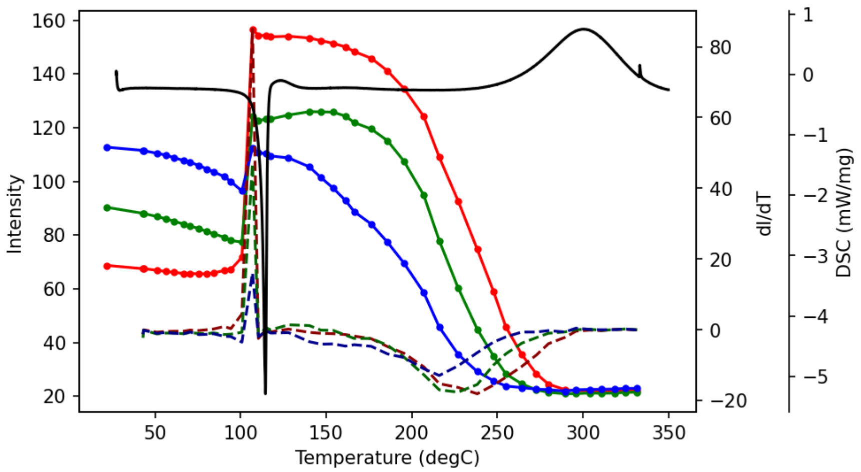

Figure S12: Calibration of 10,12 docosadiyndioic acid (DCDA). The solid lines with 'o' markers are the RGB channels, dashed lines indicate derivatives of the RGB channels. The solid black line is the DSC curve.

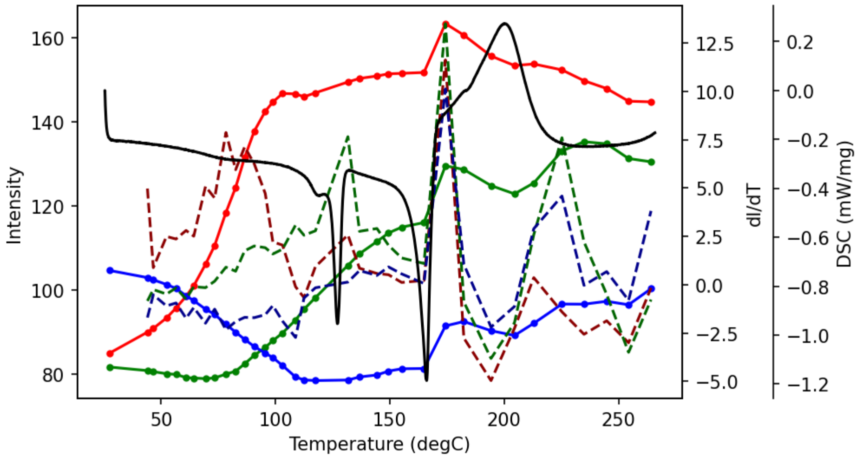

Figure S13: Calibration of 4-boronic acid functionalized PCDA (4BA-PCDA). The solid lines with 'o' markers are the RGB channels, dashed lines indicate derivatives of the RGB channels. The solid black line is the DSC curve.

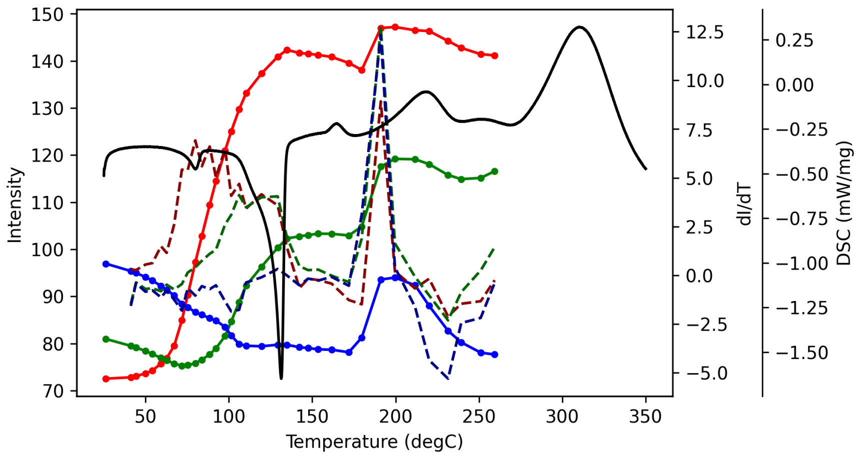

Figure S14: Calibration of 3-boronic acid functionalized PCDA (3BA-PCDA). The solid lines with 'o' markers are the RGB channels, dashed lines indicate derivatives of the RGB channels. The solid black line is the DSC curve.

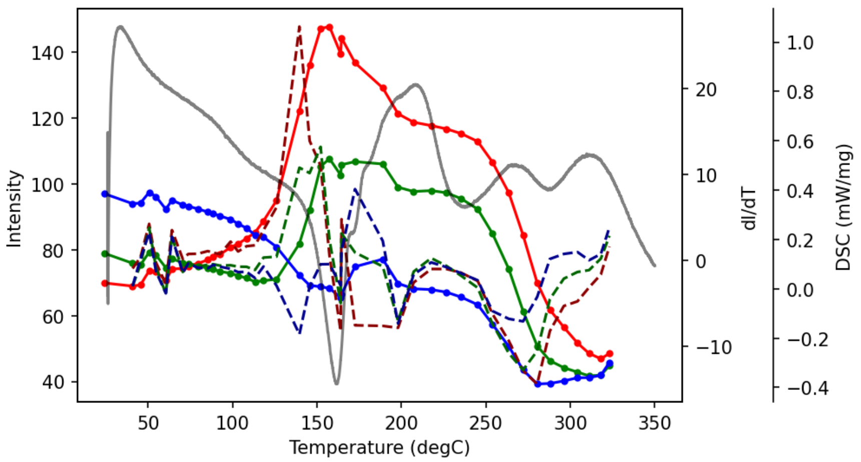

Figure S15: Calibration of 3-boronic acid functionalized PCDA (3BA-DCDA). The solid lines with 'o' markers are the RGB channels, dashed lines indicate derivatives of the RGB channels. The solid black line is the DSC curve.

S5 Temperature Tracking with PDAs

S5.1 Combustion Measurements

A 4 kW "Dwarf" wood stove from Tiny Wood Stove was used as a quiescent chamber for combustion measurements[1]. Paper and cardboard samples were mounted vertically and

imaged using a FLIR BFS-U3-63S4C-C camera (6.3 MP, 60 fps) with either a 35 mm C series lens or 75 mm DG lens (Edmund Optics). Nitrocellulose deflagrations were imaged with Phantom Miro LC121 high speed camera set to 1700 fps (588 µs, 0.9 MP) fitted with a 105mm Micro-NIKKOR lens. Acquisition and analysis software was written in Python utilizing Pillow, OpenCV, pycromanager, and FIJI packages [2, 3, 4, 5].

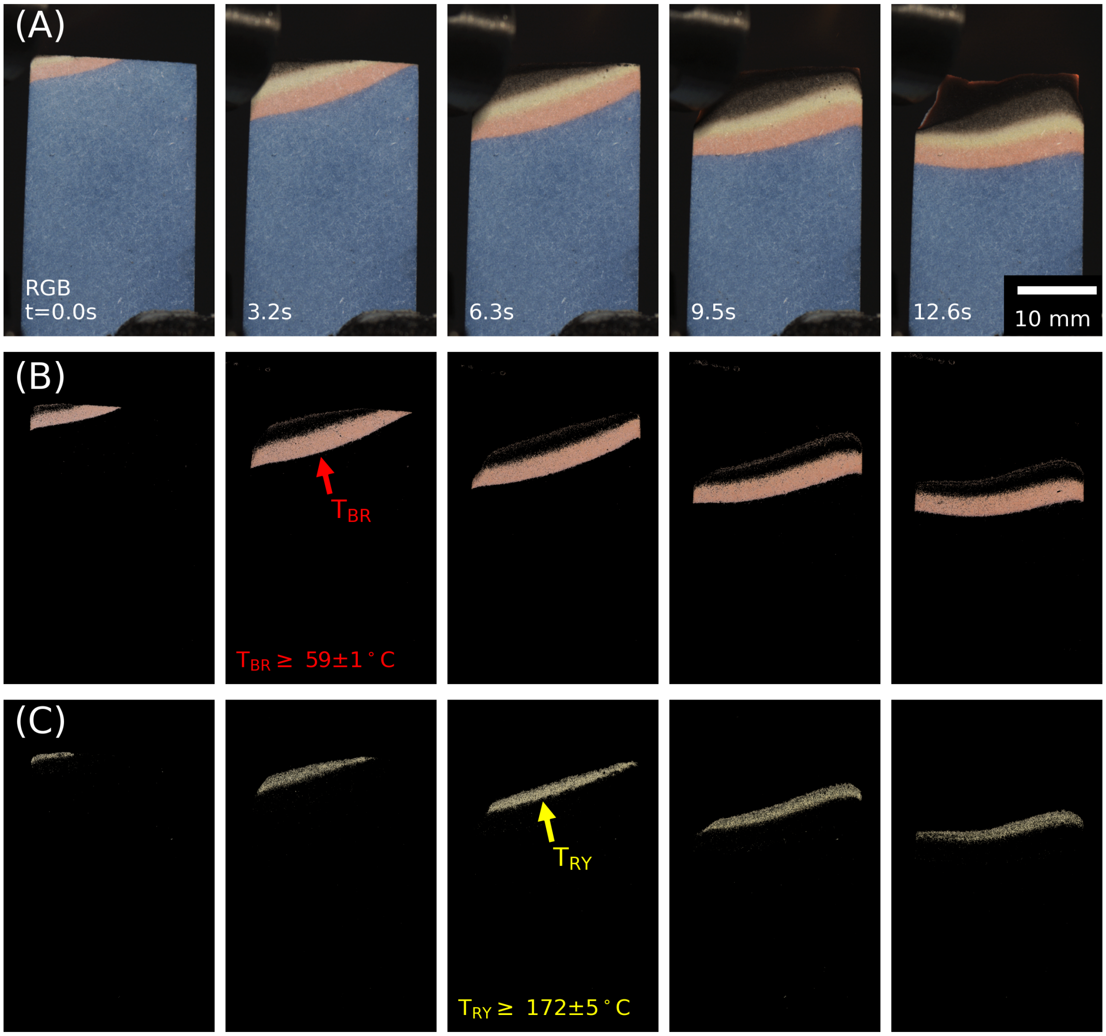

Figure S16: Tracking red and yellow phase PDA (PCDA) during combustion with HSV thresholding. (A) Original color images (B) Thresholded images to track the red phase (T $\geq$ 59 $\pm$ 1 °C), the arrow indicates the blue to red transition temperature. (C) Thresholded image series to track the yellow phase (T $\geq$ 172 $\pm$ 5 °C). The arrow indicates the interface between the yellow and red phase. Video 1 shows this progression.

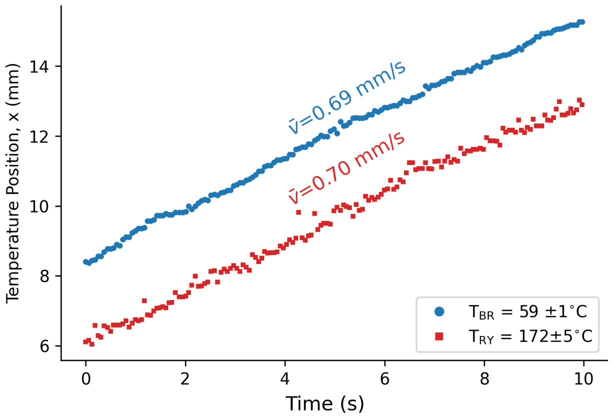

Figure S17: Direct tracking of temperature is obtained via image thresholding, Fig. S16 and recording the position of the blue-to-red and red-to-yellow interfaces. (A) Position of T_{BR} and T_{RY} for a vertical image slice as a function of time, $\bar{v}$ indicates the average velocity $(\frac{dx}{dt})$ of the two temperature bands. This process is shown in Video 1.

S5.2 Tracking a very fine temperature gradient, 3BA-DCDA

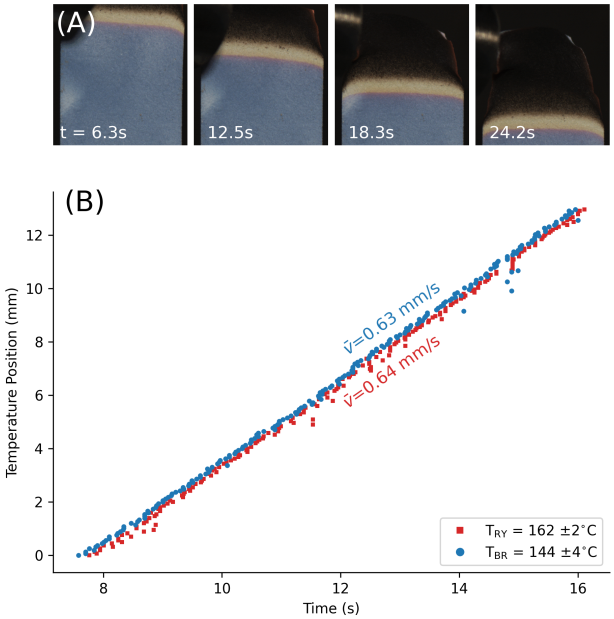

Figure S18: (A) Select images of combustion of paper coated in 3BA-PCDA in opposed flow conditions with a $T_{BR} = 144 \pm 4$ and $T_{RY} = 162 \pm 2$. (B) Vertical slice of the images showing the progression of the temperature bands, $\bar{v}$ indicates the average velocity of the temperature bands.

S6 Demonstration: Paper Combustion

S6.1 Paper coated in DCDA

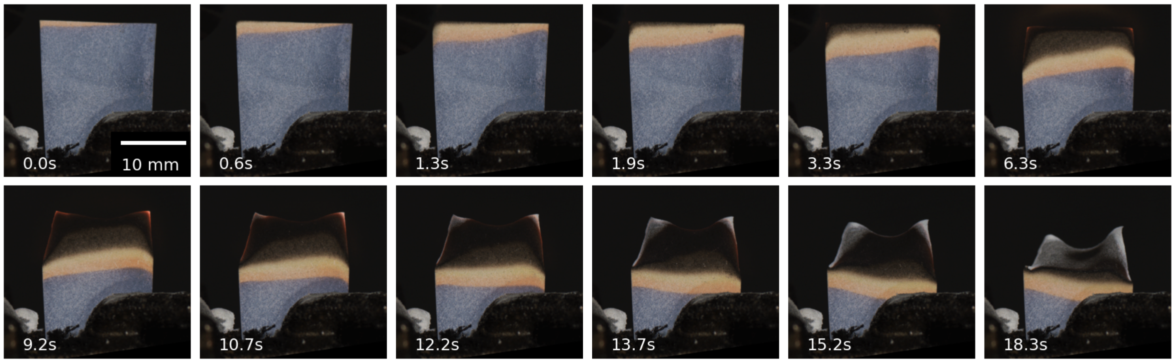

Figure S19: Paper coated in DCDA burning, T_{BR} ≈108, T_{RY} ≈209

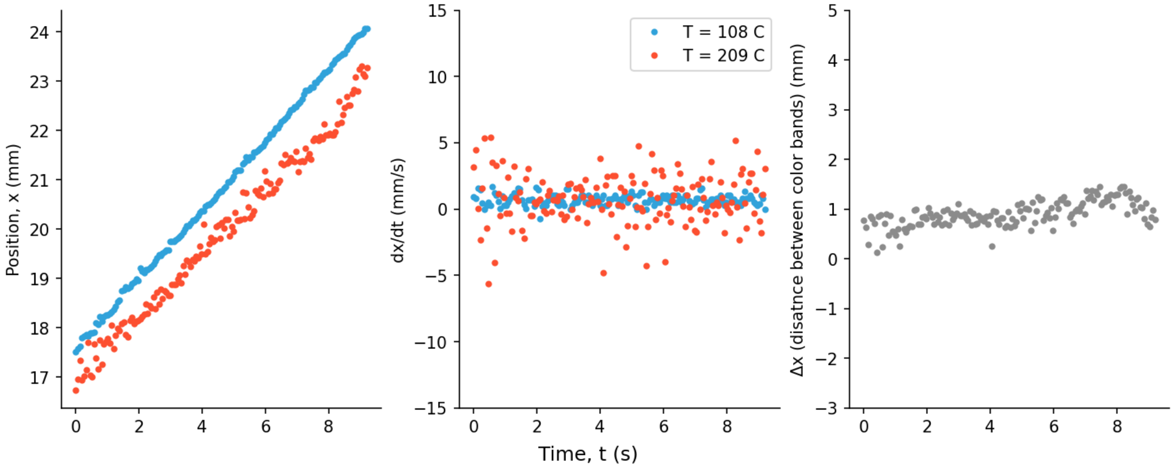

Figure S20: Tracking T_{BR} ≈108 and T_{RY} ≈209 from Fig. S19

S6.2 Opposed Flow Horizontal Combustion: PCDA

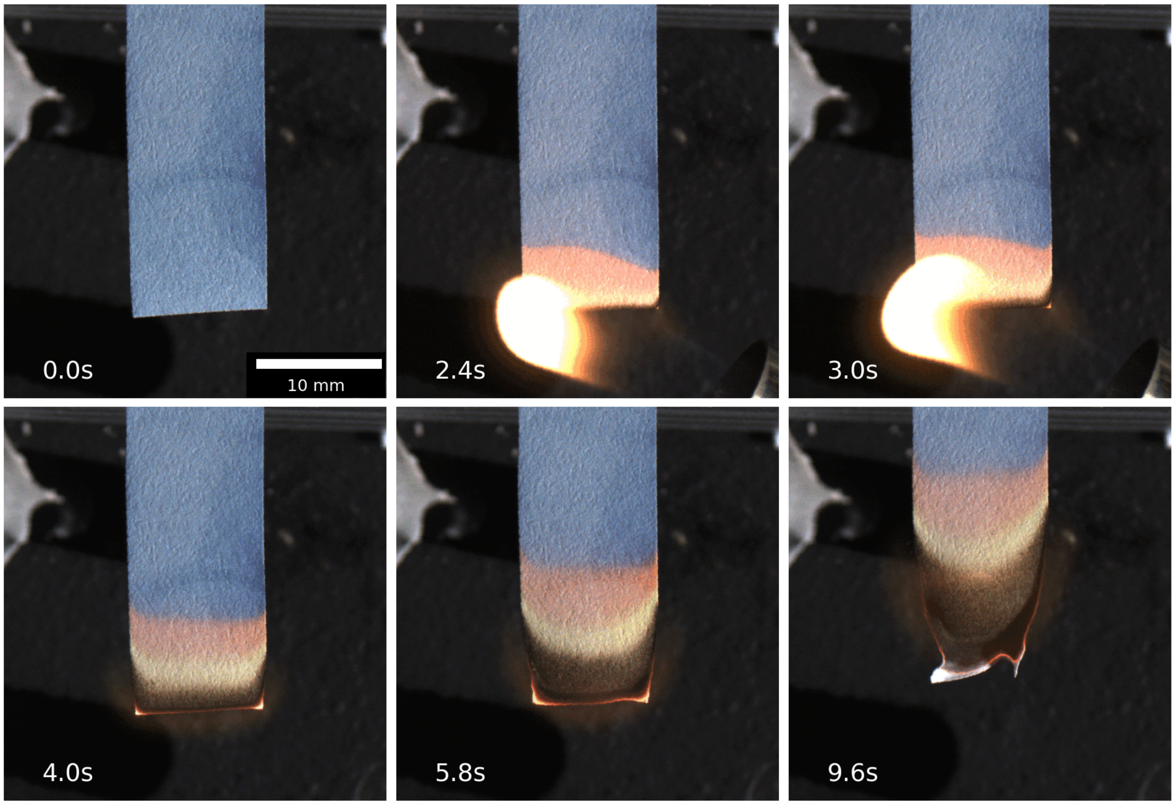

Figure S21: Horizontal combustion of paper coated in PCDA (camera is looking down on the paper) T_{BR} ≈59, T_{RY} ≈172

S6.3 Opposed Flow Horizontal Combustion: DCDA

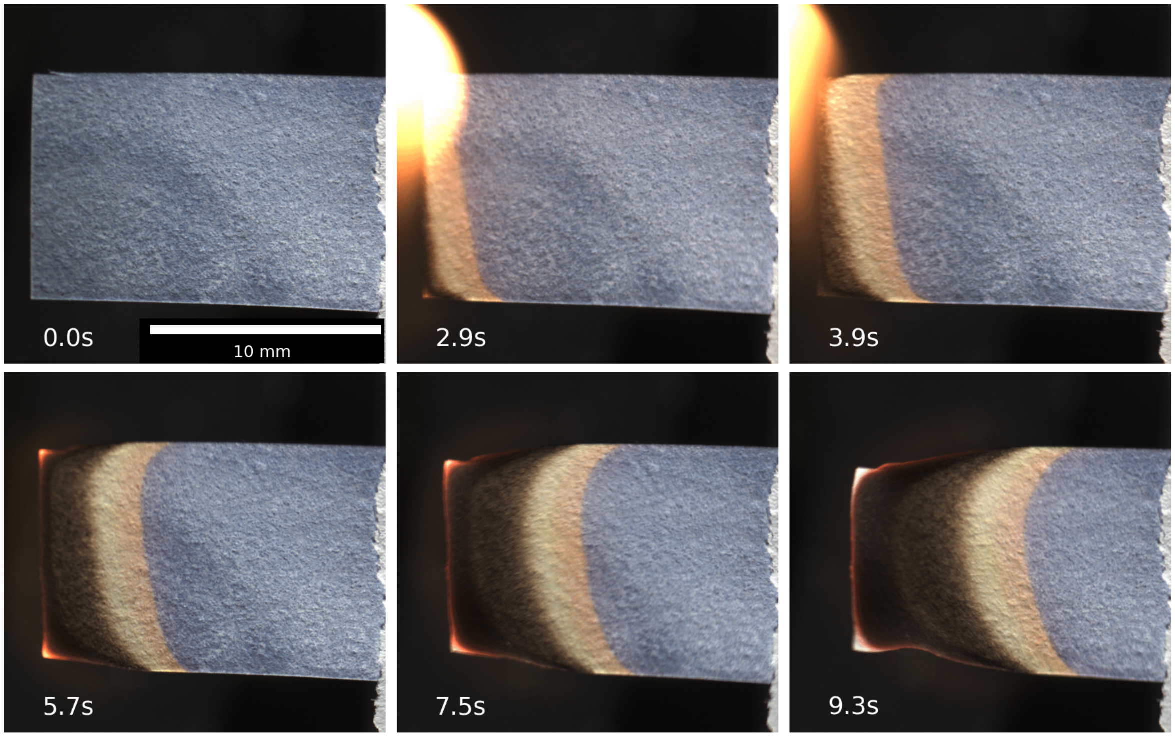

Figure S22: Horizontal combustion of paper coated in DCDA (camera is looking down on the paper) T_{BR} ≈108, T_{RY} ≈209

S6.4 Opposed Flow Horizontal Combustion: 3BA-DCDA

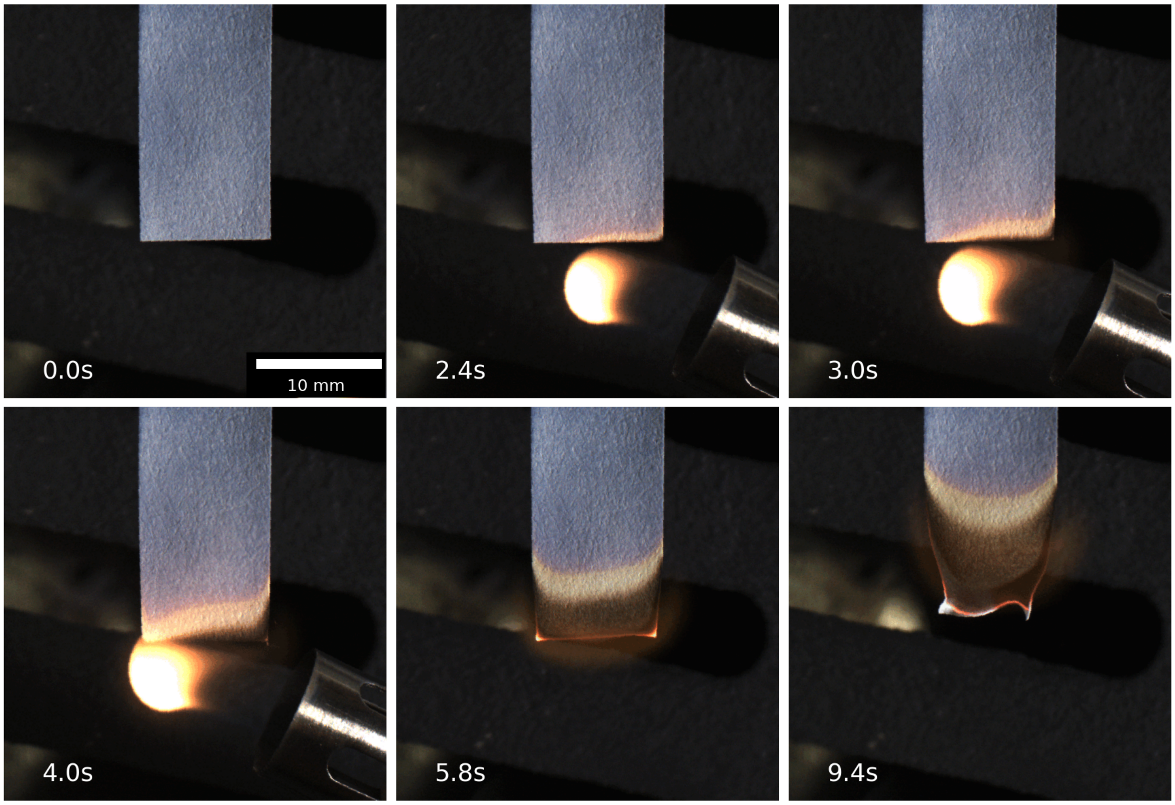

Figure S23: Horizontal combustion of paper coated in 3BA-DCDA (camera is looking down on the paper) $T_{BR} \approx 144$, $T_{RY} \approx 162$

S7 Demonstration: Nitrocellulose

All images here were captured with a Miro LC121 with 105mm Nikon Macro lens at 1700 fps.

S7.1 Guncotton coated in PCDA

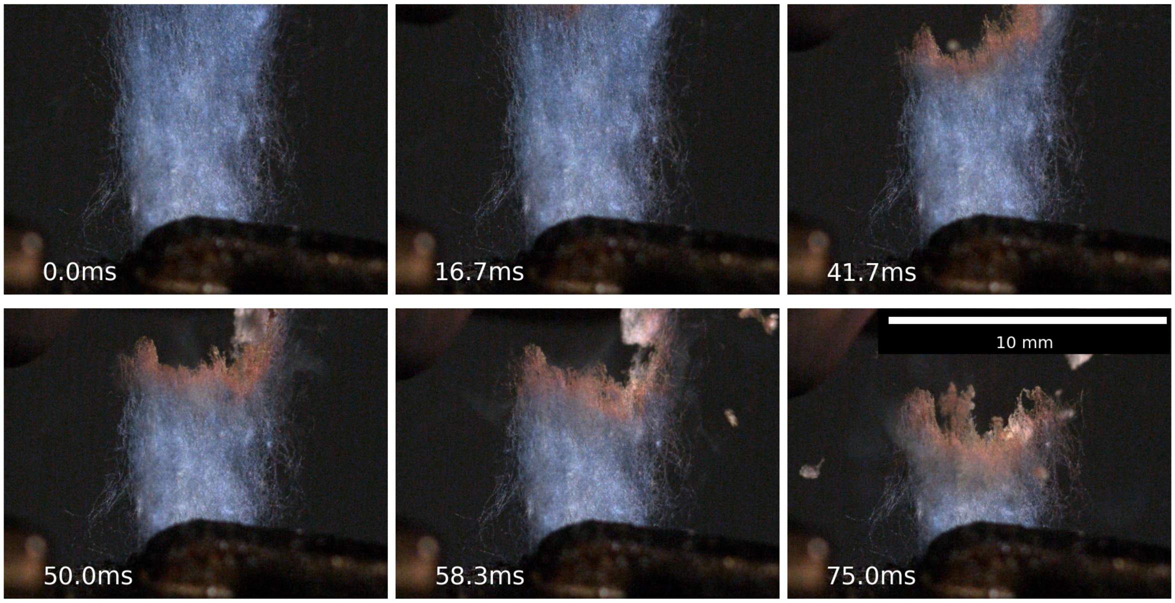

Figure S24: Horizontal combustion of nitrated cotton coated in PCDA $T_{BR} \approx 59$, $T_{RY} \approx 172$

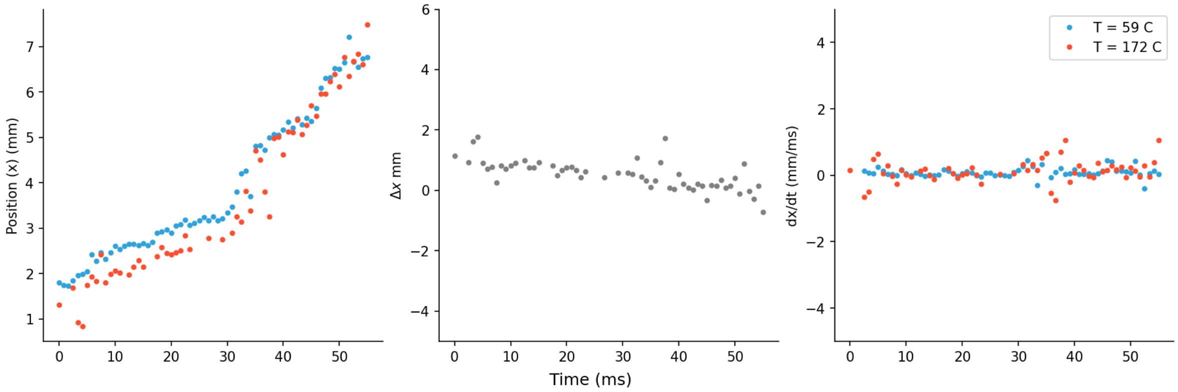

Figure S25: Tracking of the blue and red bands, $T_{BR} \approx 59$, $T_{RY} \approx 172$ from Fig. S24

S7.2 Nitrated Paper coated in PCDA

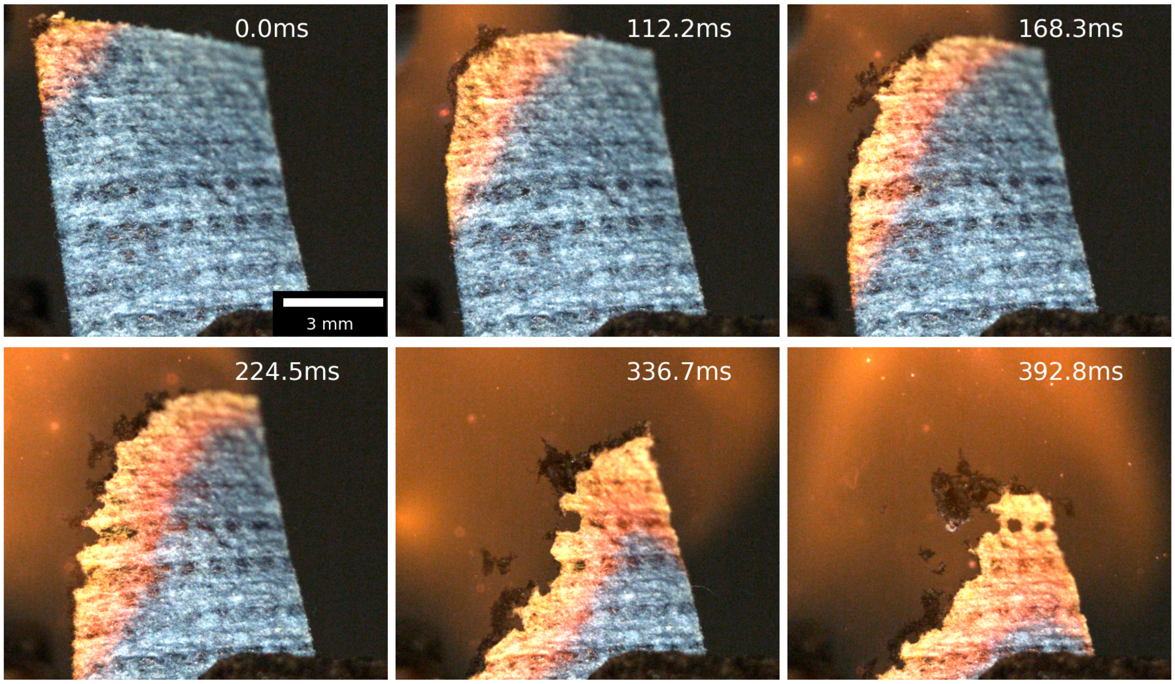

Figure S26: Combustion of nitrated cotton wipe coated in PCDA T_{BR} ≈59, T_{RY} ≈172

S7.3 Nitrated Paper coated in DCDA

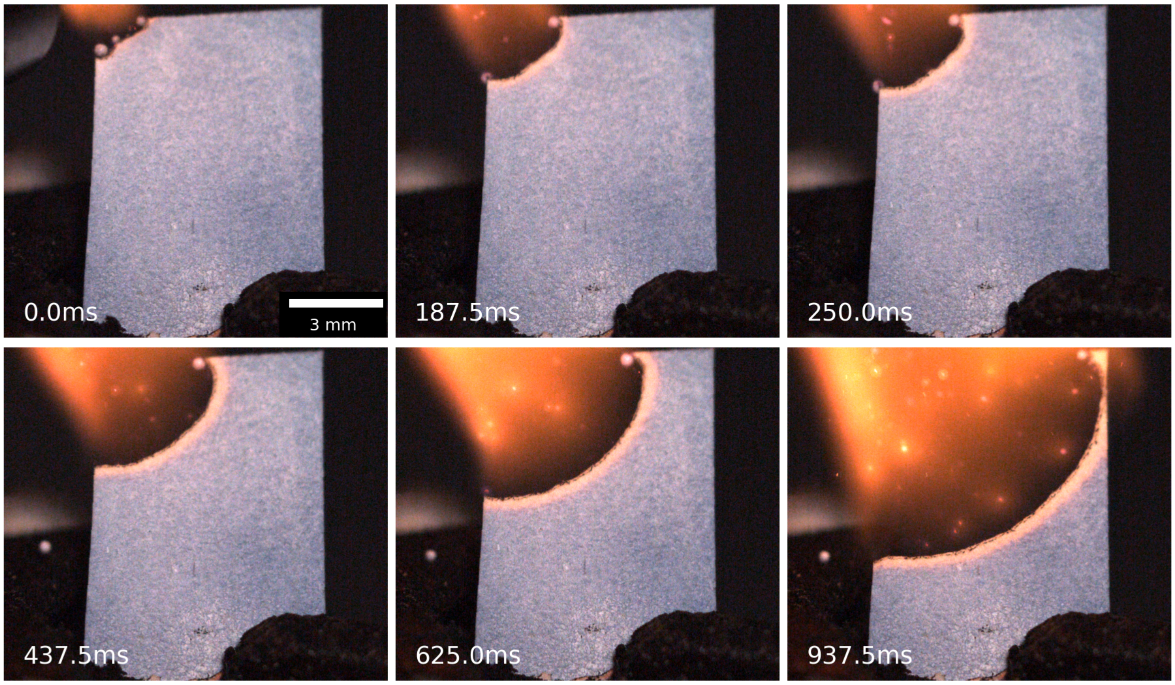

Figure S27: Combustion of nitrated cotton paper coated in DCDA T_{BR} ≈108, T_{RY} ≈209

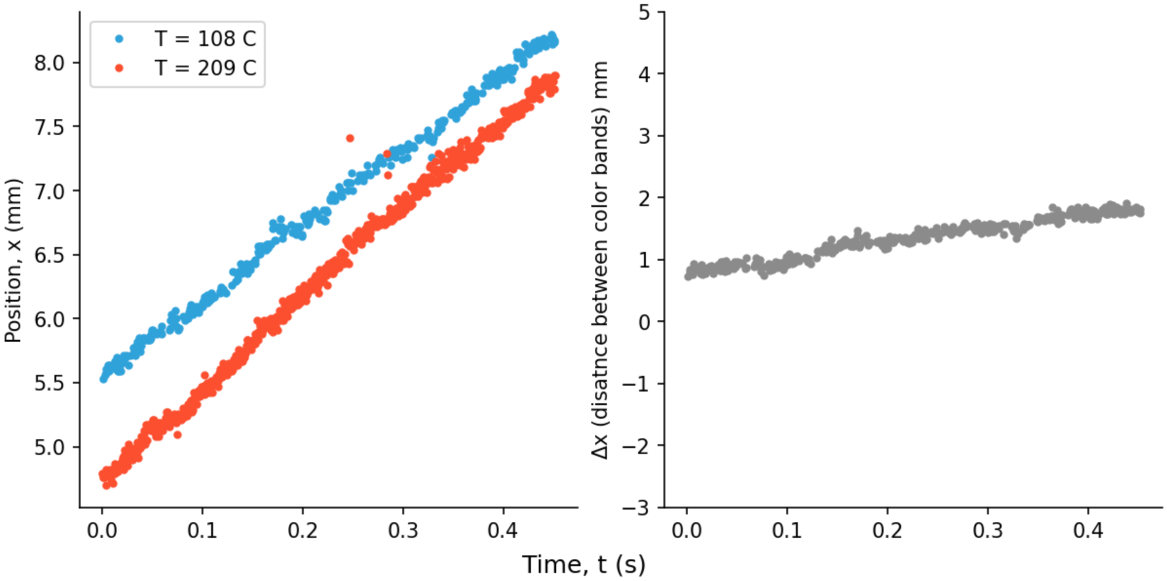

Figure S28: Tracking T_{BR} ≈108 and T_{RY} ≈209 from Fig. S27

S7.4 Nitrated Paper coated in 3BA-DCDA

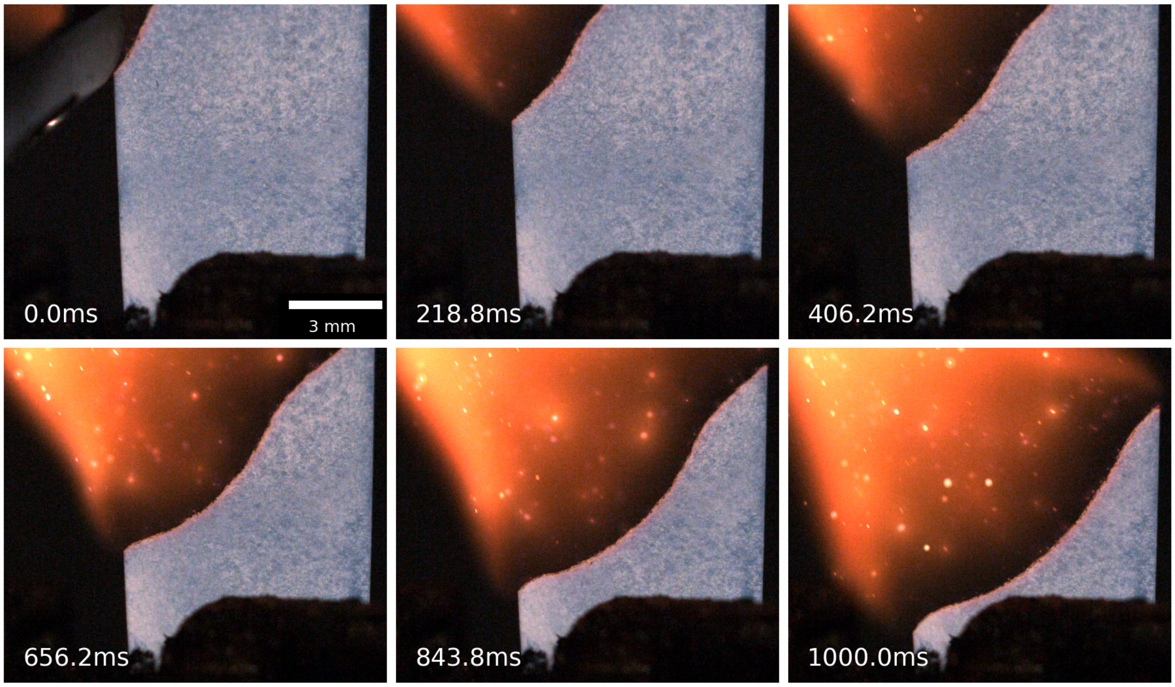

Figure S29: Combustion of nitrated cotton paper coated in 3BA-DCDA T_{BR} ≈144, T_{RY} ≈162. The yellow band is very narrow in this example.

S8 Demonstration: Comb Combustion

	Calibrated ΔT °C	$\approx \Delta x$(T_{BR} - T_{RY}) mm
TCDA	108	1.7
PCDA	112	1.1
DCDA	102	0.4
3BA-DCDA	17	0.2

Table S1: Temperature difference (ΔT) between T_{BR} and T_{RY} and distance (Δx) between $_{BR}$ and T_{RY} from comb burns (Figure in main text)

S8.1 Temperature as a Function of Approximate Location

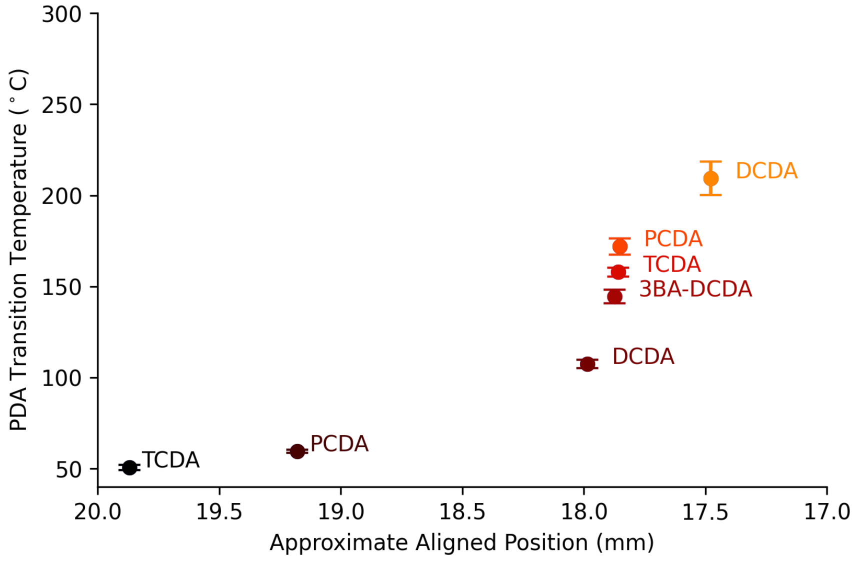

Figure S30: Position of the temperature bands on each tine of a comb burn. The actual positions had to be shifted as ignition time was not perfect and is thus a rough approximation to demonstrate the temperature gradients observed in the comb burns.

S8.2 Horizontal Combustion

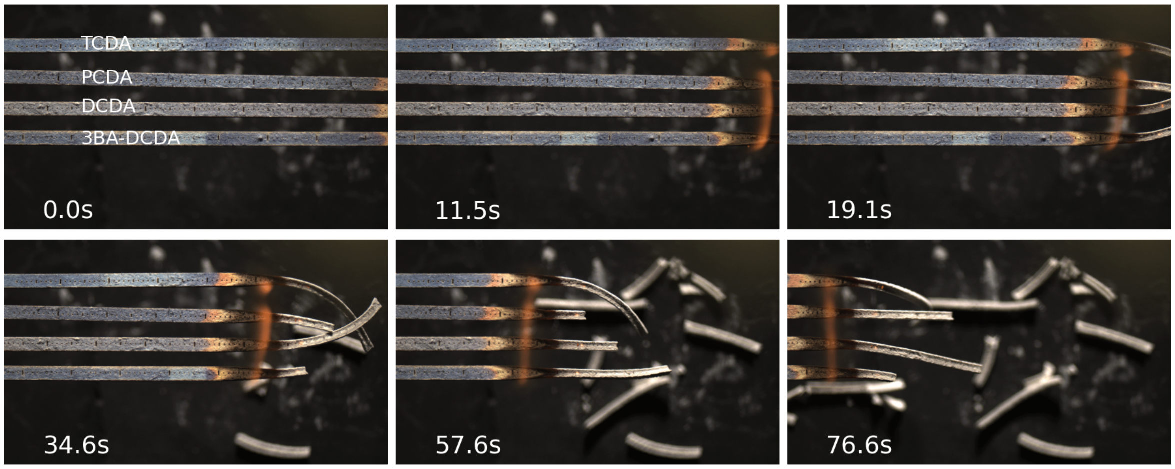

Figure S31: Cardboard comb combustion burning horizontally (camera is looking down)

S9 Demonstration: Smoldering

S9.1 Incense Stick

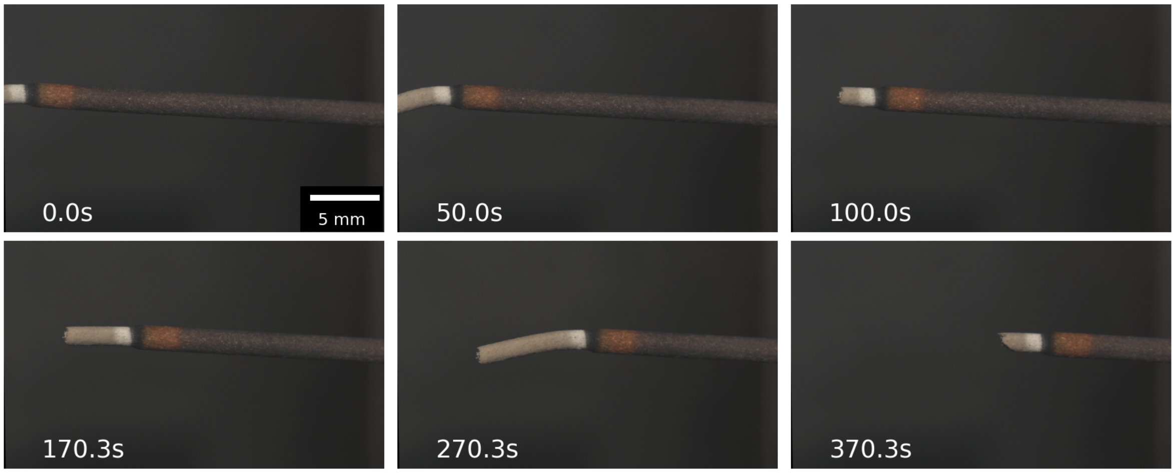

Figure S32: Smoldering incense stick coated in TCDA

S10 ^{1}H NMR of each synthesized diacetylene

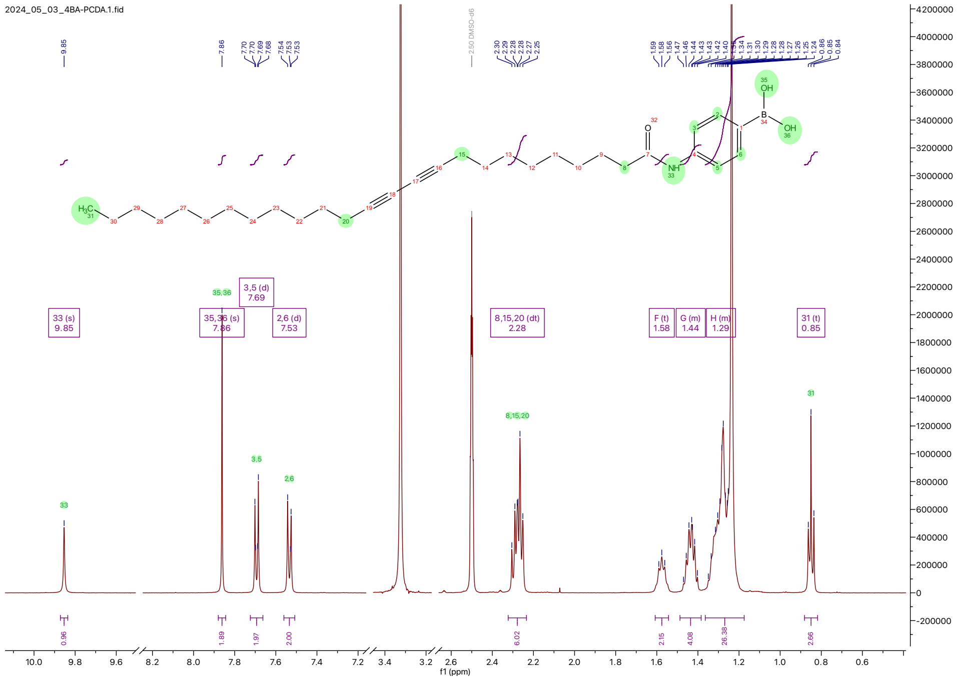

Figure S33: 4BA-PCDA Proton NMR

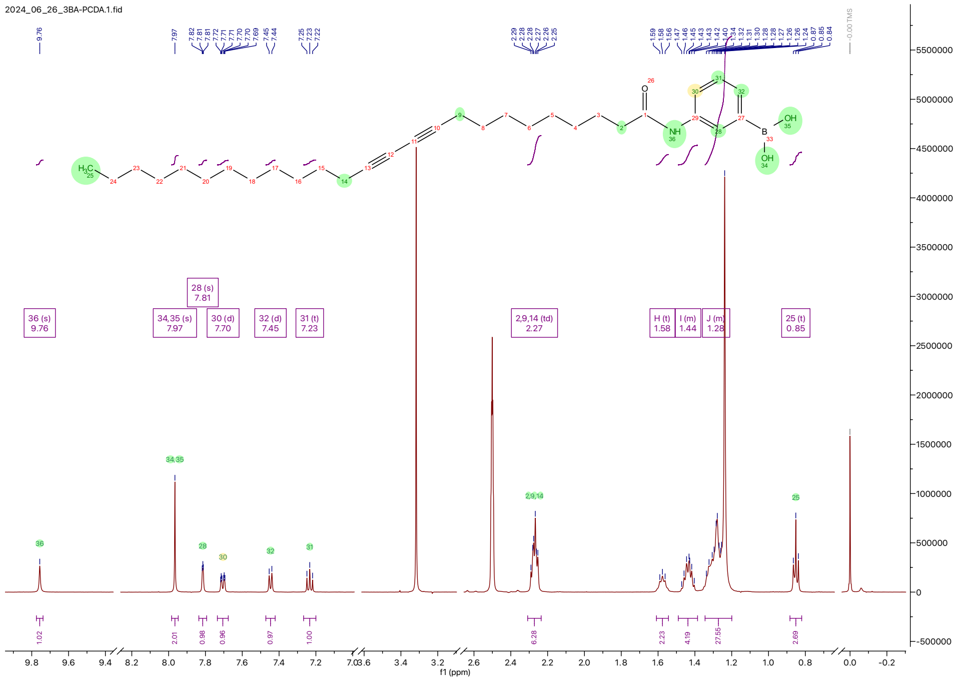

Figure S34: 3BA-PCDA Proton NMR

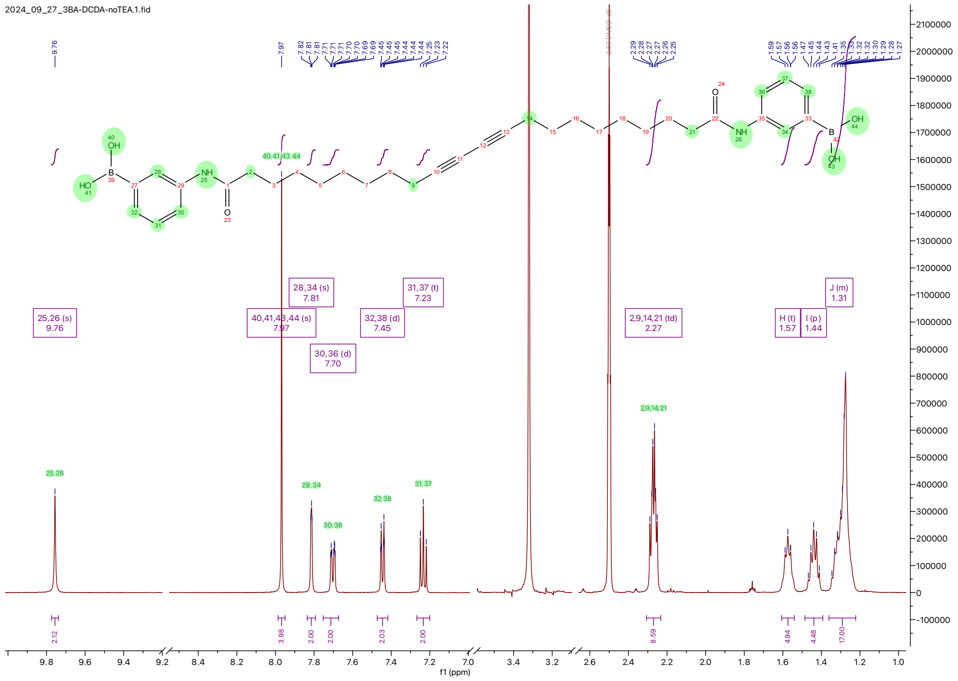

Figure S35: 3BA-DCDA Proton NMR

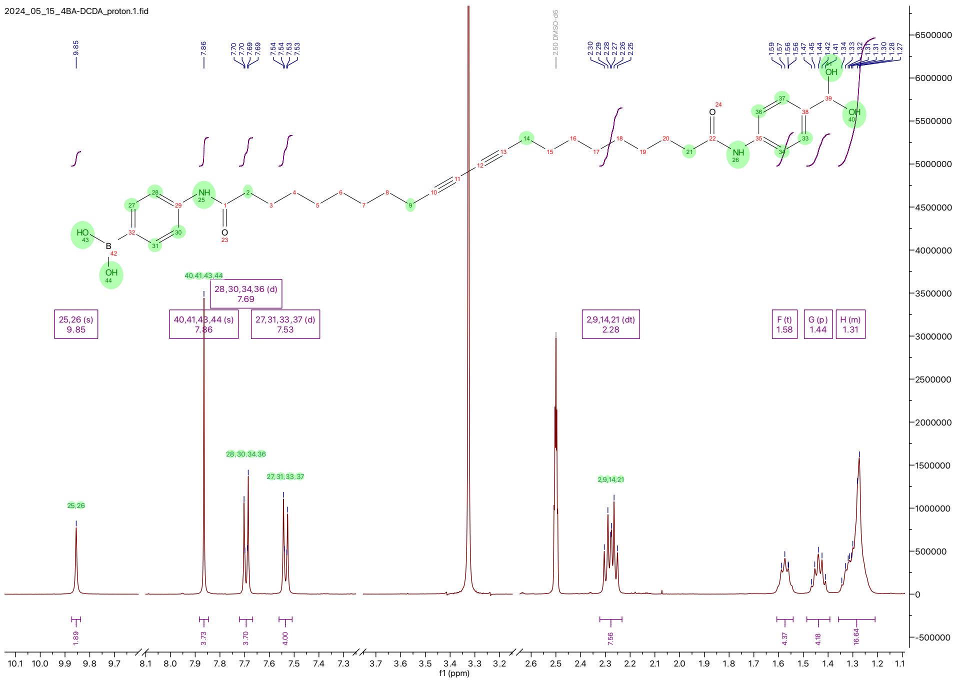

Figure S36: 4BA-DCDA Proton NMR

References

[1] Tiny Wood Stove. https://www.tinywoodstove.com/, April 2024.

[2] G. Bradski. The OpenCV library. *Dr. Dobb's Journal of Software Tools*, 2000.

[3] Alex Clark. Pillow (PIL fork) documentation, 2015.

[4] Johannes Schindelin, Ignacio Arganda-Carreras, Erwin Frise, Verena Kaynig, Mark Longair, Tobias Pietzsch, Stephan Preibisch, Curtis Rueden, Stephan Saalfeld, Benjamin Schmid, Jean-Yves Tinevez, Daniel James White, Volker Hartenstein, Kevin Eliceiri, Pavel Tomancak, and Albert Cardona. Fiji: An open-source platform for biological-image analysis. *Nature Methods*, 9(7):676–682, July 2012. ISSN 1548-7091, 1548-7105. doi: 10.1038/nmeth.2019.

[5] Henry Pinkard, Nico Stuurman, Ivan E. Ivanov, Nicholas M. Anthony, Wei Ouyang,

Bin Li, Bin Yang, Mark A. Tsuchida, Bryant Chhun, Grace Zhang, Ryan Mei, Michael Anderson, Douglas P. Shepherd, Ian Hunt-Isaak, Raymond L. Dunn, Wiebke Jahr, Saul Kato, Loïc A. Royer, Jay R. Thiagarajah, Kevin W. Eliceiri, Emma Lundberg, Shalin B. Mehta, and Laura Waller. Pycro-Manager: Open-source software for customized and reproducible microscope control. *Nature Methods*, 18(3):226–228, March 2021. ISSN 1548-7091, 1548-7105. doi: 10.1038/s41592-021-01087-6.
